# Glucose‐Responsive PAGR1‐Regulated Skeletal Muscle Gene Program Controls Systemic Glucose Homeostasis and Hepatic Metabolism

**DOI:** 10.1002/advs.202502763

**Published:** 2025-07-24

**Authors:** Chenyun Ding, Yuhuan Jia, Lin Liu, Wen Wang, Danxia Zhou, Zheng Zhou, Likun Yang, Xinyi Chen, Di Chen, Yan Mao, Liwei Xiao, Cai‐Zhi Liu, Zhen‐Yu Du, Yujing Yin, Qiqi Guo, Zongchao Sun, Kai Ge, Tingting Fu, Hai‐Long Piao, Zhenji Gan

**Affiliations:** ^1^ The State Key Laboratory of Pharmaceutical Biotechnology and MOE Key Laboratory of Model Animal for Disease Study Division of Spine Surgery Department of Orthopedic Surgery Nanjing Drum Tower Hospital Affiliated Hospital of Nanjing University Medical School Chemistry and Biomedicine Innovation Center (ChemBIC) Model Animal Research Center Medical School of Nanjing University Nanjing University Nanjing 210061 China; ^2^ State Key Laboratory of Phytochemistry and Natural Medicines Dalian Institute of Chemical Physics Chinese Academy of Sciences Dalian 116023 China; ^3^ Department of Endocrinology and Metabolism Shanghai Diabetes Institute Shanghai Clinical Center for Diabetes Shanghai Key Laboratory of Diabetes Mellitus Shanghai Sixth People's Hospital Affiliated to Shanghai Jiao Tong University School of Medicine Shanghai 200233 China; ^4^ LANEH School of Life Sciences East China Normal University Shanghai 200241 China; ^5^ Adipocyte Biology and Gene Regulation Section National Institute of Diabetes and Digestive and Kidney Diseases National Institutes of Health Bethesda MD 20892 USA; ^6^ Department of Biochemistry & Molecular Biology School of Life Sciences China Medical University Shenyang 110122 China

**Keywords:** glucose homeostasis, hepatic metabolism, obesity, skeletal muscle

## Abstract

Chronic hyperglycemia, a defining feature of type 2 diabetes (T2D) and related metabolic disorders, exacerbates insulin resistance and impairs muscle glucose utilization, contributing to systemic metabolic dysfunction. While skeletal muscle is the primary site for postprandial glucose uptake and plays a pivotal role in maintaining whole‐body glucose homeostasis, the molecular mechanisms by which hyperglycemia induces maladaptive responses in muscle remain poorly understood. Here, PAXIP1‐associated glutamate‐rich protein 1 (PAGR1) is identified as a glucose‐responsive regulator in skeletal muscle, whose expression is induced by high glucose levels and modulates systemic glucose homeostasis and hepatic metabolism. Using muscle‐specific PAGR1‐knockout mice, it is demonstrated that PAGR1 deficiency enhances insulin signaling, promotes glucose transporter 4 (GLUT4) translocation, and increases muscle glucose uptake and utilization. Mechanistically, PAGR1 directly activates the expression of TBC1 Domain Family Member 4 (TBC1D4), a RAB GTPase Activating Protein (RabGAP) known to negatively regulate GLUT4 translocation. Importantly, muscle‐specific deletion of PAGR1 protects against high‐fat‐diet‐induced insulin resistance and hepatic steatosis. These findings establish PAGR1 as a critical mediator of muscle glucose sensing and utilization, positioning it as a potential target for therapeutic strategies aimed at mitigating glucotoxicity and preventing metabolic diseases such as T2D.

## Graphical abstract

The study reveals that PAXIP1‐associated glutamate‐rich protein 1 (PAGR1), a glucose‐responsive regulator in skeletal muscle, modulates systemic glucose homeostasis and hepatic metabolism. Muscle‐specific PAGR1 deletion enhances insulin signaling, promotes glucose transporter 4 (GLUT4) translocation, and protects against high‐fat‐diet‐induced insulin resistance and hepatic steatosis. These findings highlight PAGR1 as a potential therapeutic target for mitigating metabolic disorders such as type 2 diabetes.

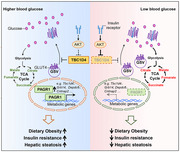

## 1 Introduction

Chronic hyperglycemia, a hallmark of type 2 diabetes (T2D) and other metabolic disorders, poses significant challenges due to its contribution to glucose toxicity, which exacerbates insulin resistance and impairs insulin secretion.^[^ ^1^ ^]^ The prolonged exposure to high glucose levels has been linked to a spectrum of complications, including microvascular damage, such as retinopathy, neuropathy, and nephropathy, as well as cardiovascular diseases and nonalcoholic liver diseases.^[^ ^2^ ^]^ As the principal site of glucose uptake and utilization, skeletal muscle plays a central role in maintaining systemic glucose homeostasis.^[^ ^3^ ^]^ Skeletal muscle's capacity for glucose uptake and utilization can be enhanced through exercise training, which improves both insulin‐dependent and insulin‐independent glucose uptake.^[^ ^3^, ^4^ ^]^ These adaptations enhance systemic insulin sensitivity, reduce the risk of metabolic syndrome, and mitigate liver fat accumulation. Conversely, defects in muscle glucose uptake and utilization systems, especially under chronic hyperglycemia, are central to the development of insulin resistance.^[^ ^5^ ^]^ The severity of muscle insulin resistance is closely related to the level of hyperglycemia, indicating a feedback loop that exacerbates metabolic dysfunction.

Skeletal muscle accounts for 60–80% of insulin‐stimulated glucose uptake, and the insulin‐sensitive glucose transporter 4 (GLUT4) mediates this process. Insulin stimulates glucose uptake in muscle through the activation of a complex signaling cascade that includes the insulin receptor, Insulin Receptor Substrate 1 (IRS‐1), Phosphatidylinositol‐3‐kinase (PI3K) and AKT Serine/Threonine Kinase (AKT).^[^ ^2^, ^5^ ^]^ This pathway leads to the phosphorylation of downstream targets such as AS160 (TBC1D4, TBC1 Domain Family Member 4), which modulates the activity of the Rab GTPase, facilitating GLUT4 movement to the plasma membrane.^[^ ^6^ ^]^ In insulin‐resistant states, muscle cells often show defective insulin‐stimulated GLUT4 translocation to the cell surface, leading to reduced glucose uptake.^[^ ^7^ ^]^ This defect occurs even when overall GLUT4 protein levels remain normal, as seen in individuals with T2D and certain rodent models.^[^ ^8^ ^]^ The PI3K–AKT signaling pathway is the well‐characterized routes controlling glucose uptake and metabolism in skeletal muscle, and this pathway's efficiency is modulated by a variety of regulatory proteins and signaling events.^[^ ^1^, ^9^ ^]^ Beyond classical insulin signaling mechanisms, emerging evidence highlights that skeletal myocytes can directly sense glucose and trigger hormone‐independent activation of AKT.^[^ ^10^ ^]^ This glucose‐sensing pathway works alongside insulin to enhance muscle glucose uptake and support systemic glucose homeostasis. Hyperglycemia leads to downregulation and dysfunction of the skeletal muscle glucose transport system. This defect impairs glucose transport and glycogen synthesis, exacerbating insulin resistance.^[^ ^11^ ^]^ The inability of insulin to promote muscle glucose uptake effectively contributes to hyperglycemia and further glucose toxicity. However, how hyperglycemia triggers maladaptive responses leading to impaired muscle glucose utilization and systemic metabolic dysfunction remains unclear.

Epigenomic regulation is crucial for gene–environment interactions and is increasingly recognized as a key factor in the pathogenesis of T2D.^[^ ^12^ ^]^ The dynamic nature of epigenetic modifications allows cells to respond reversibly to environmental cues, making it a critical mechanism for controlling gene expression in response to metabolic changes. PAXIP1‐associated glutamate‐rich protein 1 (PAGR1), also known as PA1, was first identified as part of the PTIP complex within the MLL3/MLL4 histone H3K4 methyltransferase complexes.^[^ ^13^ ^]^ PAGR1 has been implicated in multiple biological processes, including embryonic development, adipogenesis, DNA damage response, immunoglobulin class switching, and cancer progression.^[^ ^14^ ^]^ However, the role of PAGR1 in metabolic regulation, particularly in skeletal muscle, remains unknown.

In this study, we investigated the role of PAGR1 in skeletal muscle adaptation to hyperglycemia. We discovered that PAGR1 expression in skeletal muscle responds to glucose availability and modulates muscle glucose utilization pathways. Strikingly, muscle‐specific depletion of PAGR1 led to enhanced insulin signaling, improved glucose disposal in skeletal muscle, and protection against high‐fat‐diet (HFD)‐induced insulin resistance and hepatic steatosis. Mechanistically, the metabolic benefits associated with PAGR1 depletion were driven by increased glucose uptake and utilization in muscle, mediated by the activation of the insulin signaling pathway. Our findings further revealed that PAGR1 directly regulates TBC1D4, a key RabGAP in the insulin signaling pathway, to control GLUT4 translocation to the cell surface, facilitating glucose uptake. These insights highlight an unrecognized role of muscle PAGR1 in contributing to insulin resistance during hyperglycemia and suggest that targeting PAGR1 in skeletal muscle could be a promising therapeutic strategy for managing metabolic diseases.

## 2 Results

### 2.1 PAGR1 Levels are Regulated in Skeletal Muscle in Response to Glucose Stimuli

**Figure 1:**
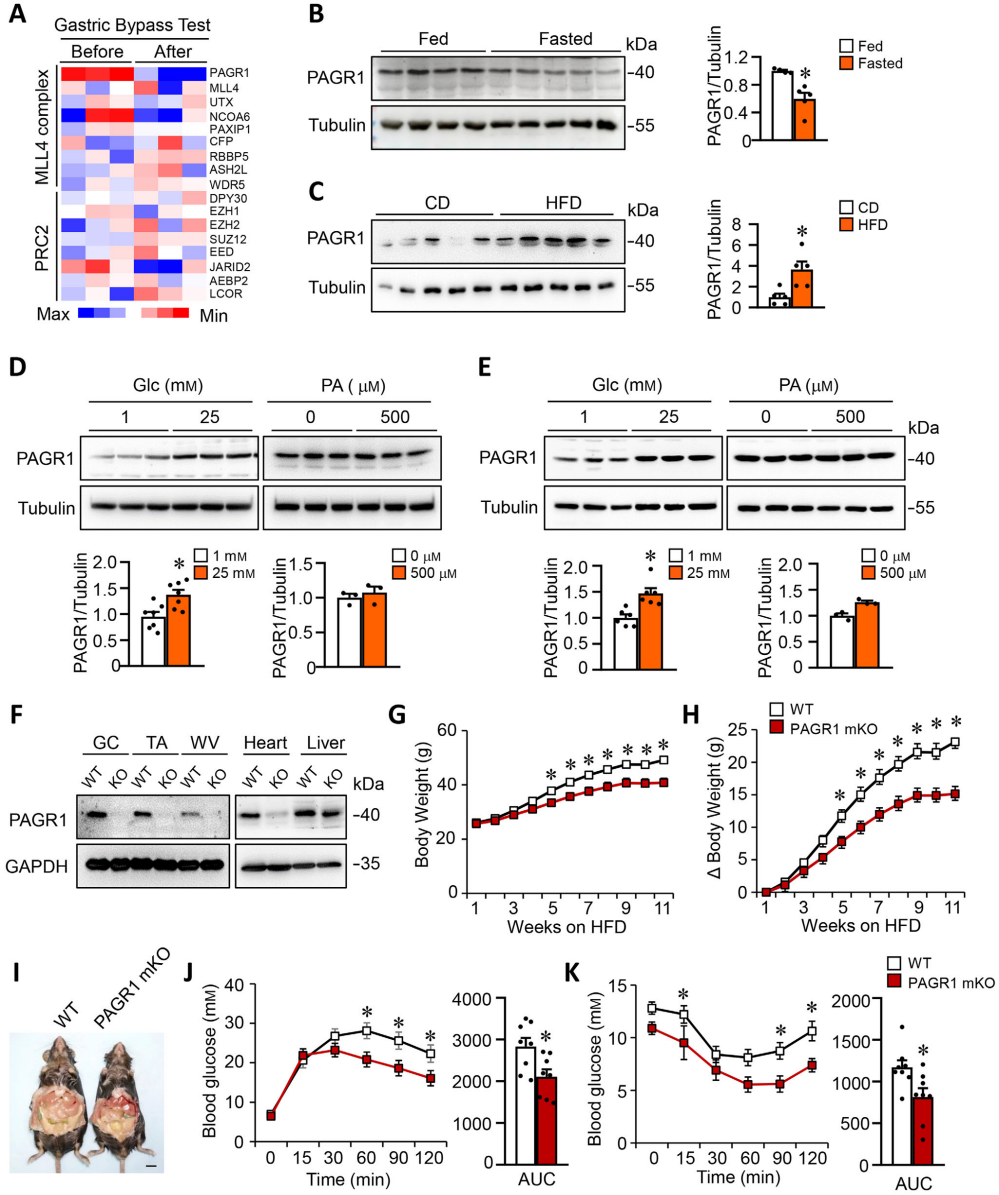
Glucose‐responsive PAGR1 regulates systemic glucose homeostasis. A) Relative expression of MLL4 complex and PRC2 subunits in human skeletal muscle tissue analyzed before and after gastric bypass surgery. Data were extracted from the GEO dataset GSE5109. B) Representative Western blot analysis of protein extracts from gastrocnemius (GC) muscles of fed and fasted WT mice using the indicated antibodies. Quantification of PAGR1/α‐TUBULIN signal ratios, normalized to the chow diet (CD, set at 1.0), is presented below the respective bands. *n* = 4–5 mice per group. C) Western blot analysis of protein extracts from white vastus lateralis (WV) muscles of WT mice fed either a normal chow diet (CD) or a high‐fat diet (HFD). Quantification of PAGR1/α‐TUBULIN signal ratios, normalized to CD (set at 1.0), is shown below the bands. *n* = 5 mice per group. D,E) Representative Western blot analysis of protein extracts from differentiated C2C12 myotubes and primary human skeletal muscle cells treated for 12 h with glucose (Glc) or palmitic acid (PA). *n* = 3–7 per group. F) Western blot analysis showing PAGR1 expression in various tissues, including GC, tibialis anterior (TA), WV muscles, heart, and liver from WT and PAGR1 muscle knockout (PAGR1 mKO) mice. G,H) Growth curves (G) and body weight gain (H) of WT and PAGR1 mKO mice. *n* = 9 mice per group. I) Representative images of WT and PAGR1 mKO mice after 16 weeks on a HFD. J) Glucose tolerance test (GTT) results. The area under the curve (AUC) for GTT is shown on the right. *n* = 8 mice per group. K) Insulin tolerance test (ITT) results. The AUC for ITT is shown on the right. *n* = 8 mice per group. All data are presented as the mean ± standard error of the mean (SEM). *p < 0.05 versus the corresponding WT controls, as determined by two‐tailed unpaired Student's *t*‐test (C, D, J, K), Mann–Whitney test (B, E) or two‐way ANOVA (G, H, J, K) followed by Fisher's least significant difference (LSD) post‐hoc test.

To investigate whether PAGR1 plays a role in skeletal muscle metabolism, we first examined gene expression profiles from muscle samples of obese patients before and after gastric bypass surgery using data from the NCBI Gene Expression Omnibus (GEO) database (accession no. GSE5109). Gastric bypass surgery is well‐documented for its effectiveness in reducing body weight and enhancing systemic metabolism. Our analysis revealed that PAGR1 was among the most significantly downregulated components of the MLL3/MLL4 complex postsurgery (**Figure** **1**A). By contrast, mRNA levels of core subunits of the polycomb‐repressive complex 2 (PRC2) exhibited minimal change (Figure 1A). To assess if muscle PAGR1 responds to nutritional and hormonal changes, we examined its protein expression in skeletal muscle from ad libitum‐fed and 16 h fasted mice. PAGR1 protein levels were significantly decreased in fasted mice compared to fed controls (Figure 1B). We then explored whether nutrient excess could modulate PAGR1 expression. Indeed, muscle PAGR1 protein levels were elevated following 2 weeks of HFD feeding (Figure 1C), demonstrating that muscle PAGR1 levels are regulated by nutrient availability.

To identify the specific nutrient signals that regulate PAGR1 expression, we treated differentiated C2C12 myotubes with metabolic factors such as glucose, insulin, and fatty acids. Notably, glucose treatment led to a substantial increase in PAGR1 protein levels, while insulin and fatty acids (palmitic acid and oleic acid) had negligible effects (Figure 1D and Figure S1A (Supporting Information)). Consistently, glucose treatment also significantly increased PAGR1 protein expression in primary human skeletal muscle cells (Figure 1E and Figure S1B (Supporting Information)). Interestingly, glucose treatment did not significantly alter PAGR1 protein levels in either HepG2 cells or primary white adipocytes (Figure S1C,D, Supporting Information), suggesting that the glucose‐responsive regulation of PAGR1 is likely specific to skeletal myocytes.

### 2.2 Muscle PAGR1 Deficiency Protects against HFD‐Induced Glucose Intolerance and Insulin Resistance

To determine the physiological significance of PAGR1 in skeletal muscle, we generated muscle‐specific PAGR1‐knockout mice (PAGR1 mKO) by crossing PAGR1‐floxed (PAGR1*^f/f^*) mice with myosin creatine kinase (MCK)‐Cre mice. PAGR1 mKO mice were born at expected Mendelian ratios and appeared phenotypically normal. Western blot analysis confirmed a significant reduction of PAGR1 protein in various muscle types of PAGR1 mKO mice compared to wild‐type (WT) littermates (Figure 1F). When fed a standard chow diet, PAGR1 mKO mice exhibited metabolic profiles similar to WT controls, including normal glucose tolerance, insulin sensitivity, and comparable adipose tissue weight and adipocyte size (Figure S2A–E, Supporting Information).

To investigate the impact of PAGR1 deficiency under metabolic stress, we subjected PAGR1 mKO and WT mice to a HFD (60% kcal from fat) for 3 months. Remarkably, PAGR1 mKO mice were protected against HFD‐induced obesity, showing significantly lower body weight and weight gain starting from the fifth week of the diet (Figure 1G,H). PAGR1 mKO mice were visibly smaller after HFD feeding compared to WT controls (Figure 1I). To further assess glucose homeostasis, we conducted glucose tolerance tests (GTTs) and insulin tolerance tests (ITTs) on HFD‐fed mice. PAGR1 mKO mice exhibited improved glucose tolerance compared to WT littermates (Figure 1J). Similarly, PAGR1 mKO mice showed heightened insulin sensitivity relative to HFD‐fed WT controls (Figure 1K). These results collectively demonstrate that muscle‐specific PAGR1 deficiency confers protection against HFD‐induced glucose intolerance and insulin resistance, highlighting PAGR1's regulatory role in skeletal muscle metabolism and systemic glucose homeostasis.

### 2.3 Muscle‐Specific Deletion of PAGR1 in PAGR1*^f/f^* Human Skeletal Actin‐Cre (Hsa‐Cre) Mice Confirms the Role of PAGR1 in Enhancing Systemic Glucose Homeostasis

**Figure 2:**
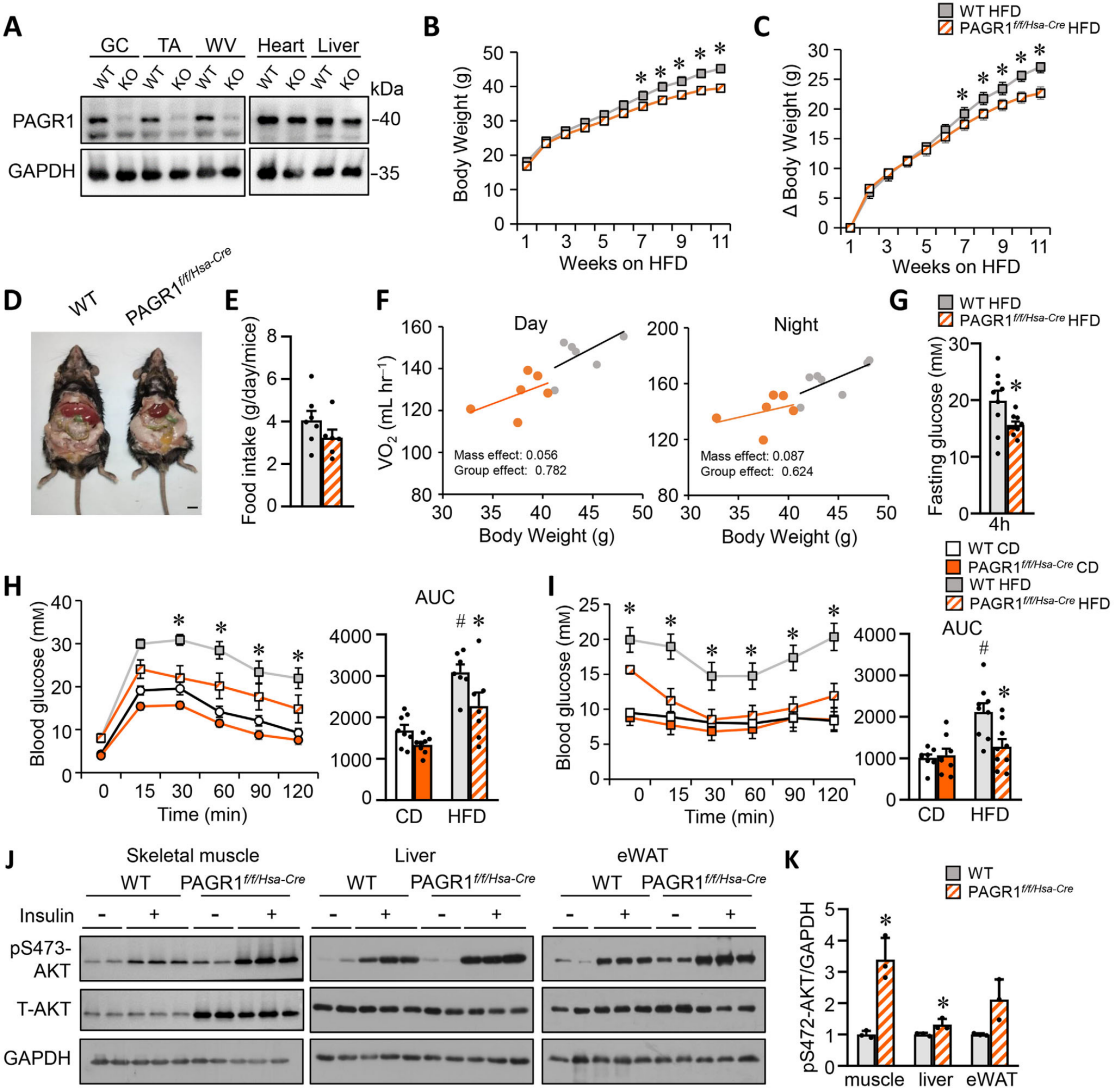
Muscle‐specific deletion of PAGR1 (PAGR1*^f/f/ Hsa‐Cre^*) protects against HFD‐induced glucose intolerance and insulin resistance. A) Western blot analysis of PAGR1 expression in the GC, TA, WV muscles, heart, and liver of WT and PAGR1*^f/f/Hsa‐Cre^* mice. B,C) Growth curves (B) and body weight gain (C) of WT and PAGR1*^f/f/Hsa‐Cre^* mice during 20 weeks of HFD feeding. 15–16 mice per group. D) Representative images of WT and PAGR1*^f/f/Hsa‐Cre^* mice after 20 weeks on a HFD. E) Food consumption data for WT and PAGR1*^f/f/Hsa‐Cre^* mice, expressed as grams consumed per gram of body weight per day. *n* = 6–7 mice per group. F) Metabolic evaluation of WT and PAGR1*^f/f/Hsa‐Cre^* mice using the comprehensive laboratory animal monitoring system (CLAMS). Regression plots illustrate metabolic parameters as a function of body weight during dark and light cycles. *n* = 6–7 mice per group. G) Fasting glucose (4 h) levels. *n* = 9 mice per group. H) GTT results, with the AUC shown on the right. *n* = 7–8 mice per group. I) ITT results, with the AUC shown on the right. *n* = 8–9 mice per group. J) Representative Western blot analysis of AKT phosphorylation at Ser473 (p‐AKT) relative to total AKT (T‐AKT) in skeletal muscle, liver, and adipose tissue 30 min after an intraperitoneal injection of insulin (2 U kg^−1^) or saline in HFD‐fed male mice. Mice were fasted overnight prior to the injection. K) Quantification of p‐AKT (S473)/T‐AKT signal ratios. *n* = 3 mice per group. All data are presented as the mean ± SEM. *p < 0.05 versus the corresponding WT controls, ^#^ *p* < 0.05 versus WT CD controls, as determined by two‐tailed unpaired Student's *t*‐test (E, H, I), Mann–Whitney test (G, K) or two‐way ANOVA (B, C, H, I) followed by Fisher's LSD post‐hoc test.

The muscle‐specific deletion of PAGR1 using MCK‐Cre previously led to reduced PAGR1 protein levels in both skeletal muscle and the heart (Figure 1F). To exclude potential confounding effects from heart‐specific PAGR1 deficiency, PAGR1*^f/f^* mice were bred with HSA‐Cre mice to achieve a targeted skeletal muscle‐specific deletion of PAGR1 without affecting the heart. As expected, PAGR1 protein expression was significantly decreased in various muscle types but remained unaltered in the heart of PAGR1*^f/f/Hsa‐Cre^* mice (**Figure** **2**A). PAGR1*^f/f/Hsa‐Cre^* mice and their WT littermates were fed either a normal chow diet or a HFD. Similar to PAGR1 mKO mice, PAGR1*^f/f/Hsa‐Cre^* mice displayed no abnormal phenotypes under a normal chow diet, including food intake, body weight, adipose tissue weight, and adipocyte size (Figure S3A–F, Supporting Information). Additionally, there were no changes in the type I muscle fibers in the gastrocnemius (GC) muscle that might influence metabolic parameters (Figure S3G, Supporting Information).

Consistent with the findings in PAGR1 mKO mice, PAGR1*^f/f/Hsa‐Cre^* mice exhibited significantly reduced weight gain after 3 months of HFD feeding compared to WT littermates, despite comparable food intake (Figure 2B–E). Indirect calorimetry analysis indicated no significant difference in oxygen consumption (VO_2_) across both light and dark cycles in PAGR1*^f/f/Hsa‐Cre^* mice (Figure 2F and Figure S4I,J (Supporting Information)). However, PAGR1*^f/f/Hsa‐Cre^* mice showed a lower respiratory exchange ratio than WT controls during HFD feeding. Fasting glucose levels were significantly lower in HFD‐fed PAGR1*^f/f/Hsa‐Cre^* mice compared to WT controls (Figure 2G). GTT and ITT further demonstrated that HFD‐fed PAGR1*^f/f/Hsa‐Cre^* mice were more tolerant to glucose challenges and more responsive to insulin stimulation than their HFD‐fed WT counterparts (Figure 2H,I). Enhanced insulin action, as evidenced by increased insulin‐stimulated AKT phosphorylation in both adipose tissue and skeletal muscle, was observed in PAGR1*^f/f/Hsa‐Cre^* mice following HFD feeding (Figure 2J). Overall, the metabolic phenotype of PAGR1*^f/f/Hsa‐Cre^* mice closely mirrors that of PAGR1 mKO mice, reinforcing the conclusion that muscle‐specific PAGR1 deficiency leads to significant protection against HFD‐induced glucose intolerance and insulin resistance.

### 2.4 Muscle Deletion of PAGR1 Prevents High‐Fat‐Diet‐Induced Hepatic Steatosis and Adipose Tissue Inflammation

**Figure 3:**
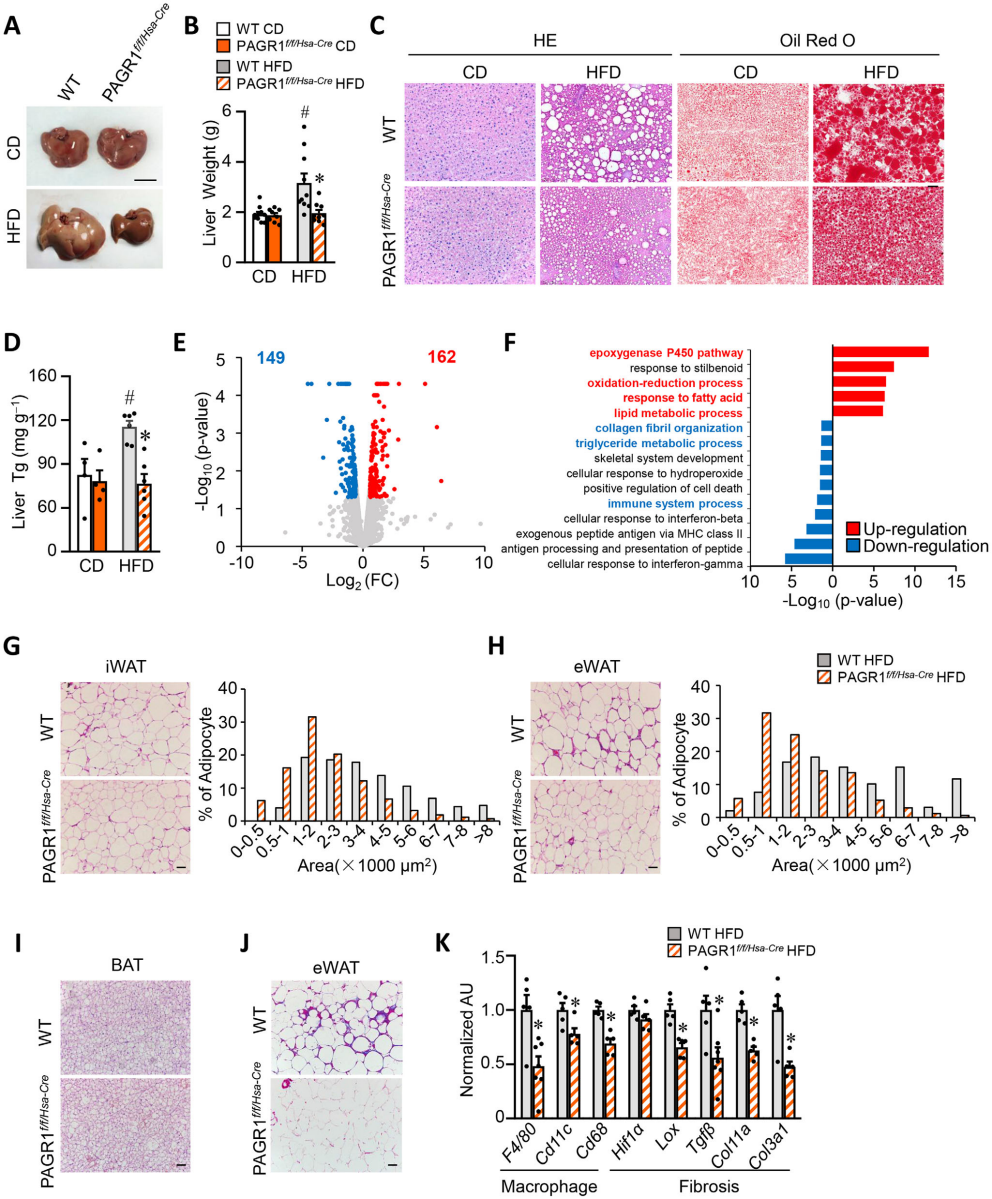
Skeletal muscle PAGR1 modulates liver and adipose tissue response to HFD. A) Representative images of livers from WT and PAGR1*^f/f/Hsa‐Cre^* mice fed with either a CD or a HFD. B) Liver weights of WT and PAGR1*^f/f/Hsa‐Cre^* mice after 16 weeks on CD or HFD. *n* = 8–10 mice per group. C) H&E and oil red O staining of liver sections from HFD‐fed WT and PAGR1*^f/f/Hsa‐Cre^* mice. The scale bar represents 50 µm. *n* = 5–7 mice per group. D) Liver triglyceride levels in HFD‐fed WT and PAGR1*^f/f/Hsa‐Cre^* mice. *n* = 4–6 mice per group. E) Volcano plot depicting fold changes versus *p*‐values for RNA‐Seq data generated from the livers of 20 week old male PAGR1*^f/f/Hsa‐Cre^* mice compared to WT littermate controls. Significantly upregulated genes are shown in red, and downregulated genes are shown in blue. *n* = 2 independent pools per group. F) GO enrichment analysis of differentially expressed genes in the liver of PAGR1*^f/f/Hsa‐Cre^* mice. G,H) Left: representative H&E staining of iWAT and eWAT from indicated male mice fed a HFD. Scale bar: 50 µm. Right: cross‐sectional areas of iWAT and eWAT were measured by ImageJ. *n* = 5 mice per group. I) H&E staining of brown adipose tissue (BAT) from WT and PAGR1*^f/f/Hsa‐Cre^* mice. *n* = 5 mice per group. J) Masson's trichrome staining of iWAT from WT and PAGR1*^f/f/Hsa‐Cre^* mice, showing fibrosis levels. The scale bar represents 50 µm. *n* = 4 mice per group. K) Quantitative reverse transcriptase‐PCR (qRT‐PCR) analysis of gene expression related to macrophages and fibrosis in eWAT from WT and PAGR1*^f/f/Hsa‐Cre^* mice following HFD feeding. *n* = 5–7 mice per group. All data are presented as the mean ± SEM. *p < 0.05 compared to the corresponding WT controls, ^#^ *p* < 0.05 versus WT CD controls, as determined by two‐tailed unpaired Student's *t*‐test (B, D) and Mann–Whitney test (K).

Emerging evidence suggests that metabolic reprogramming in skeletal muscle can influence adipose and liver metabolism. HFD‐induced obesity commonly results in triglyceride (TG) accumulation in the liver. In PAGR1*^f/f/Hsa‐Cre^* mice, PAGR1 expression was preserved in the liver (Figures 1F and 2A). Notably, gross anatomical analysis revealed markedly reduced hepatic steatosis in HFD‐fed PAGR1*^f/f/Hsa‐Cre^* mice compared to WT controls (**Figure** **3**A,B). This was accompanied by a significant reduction in liver weight in PAGR1*^f/f/Hsa‐Cre^* mice on a HFD (Figure 3A,B). Histological analysis using hematoxylin and eosin (H&E) and oil red O staining further confirmed a substantial decrease in liver fat deposition in PAGR1*^f/f/Hsa‐Cre^* mice fed with a HFD (Figure 3C). Biochemical measurements corroborated these findings, showing that the increase in hepatic TG levels observed in HFD‐fed WT mice was completely mitigated in PAGR1*^f/f/Hsa‐Cre^* mice (Figure 3D). To better understand the metabolic reprogramming in the liver of PAGR1*^f/f/Hsa‐Cre^* mice, RNA‐Seq analysis was performed on liver mRNA from PAGR1*^f/f/Hsa‐Cre^* and WT mice (Figure 3E,F). Gene ontology (GO) analysis of downregulated genes revealed significant enrichment in processes related to the immune system, triglyceride metabolism, and collagen fibril organization. Conversely, pathways associated with epoxygenase P450 and oxidation–reduction were significantly enriched among upregulated genes (Figure 3F). These findings suggest that muscle‐specific PAGR1 deletion leads to protection from HFD‐induced hepatic steatosis.

Adipose tissue metabolism was also assessed in PAGR1*^f/f/Hsa‐Cre^* mice. Histological analysis also showed smaller adipocytes in iWAT and eWAT between HFD‐fed PAGR1*^f/f/Hsa‐Cre^* mice and WT controls (Figure 3G–H). Increased macrophage infiltration and fibrosis in WAT are known markers of obesity‐related inflammation. Masson's trichrome staining revealed a significant reduction in HFD‐induced fibrosis in WAT from PAGR1*^f/f/Hsa‐Cre^* mice (Figure 3J). Additionally, the expression of macrophage markers (e.g., *F4/80*, *Cd68*, and *Cd11c*) and profibrotic genes (e.g., *Hif1a*, *Lox*, and *Tgfβ*) was markedly reduced in the WAT of HFD‐fed PAGR1*^f/f/Hsa‐Cre^* mice compared to WT controls (Figure 3K). These findings collectively suggest that PAGR1‐dependent metabolic programming in skeletal muscle plays a crucial role in interorgan metabolic communication, thereby mitigating obesity‐induced hepatic steatosis and adipose tissue inflammation.

Consistent with observations in PAGR1*^f/f/Hsa‐Cre^* mice, HFD‐fed PAGR1 mKO mice also exhibited reduced hepatic steatosis compared to WT controls (Figure S5A–D, Supporting Information). PAGR1 mKO mice also demonstrated decreased adipocyte size and fibrosis in adipose tissue relative to WT controls (Figure S5E,F, Supporting Information). Masson's trichrome staining confirmed significantly reduced fibrosis in the adipose tissue of HFD‐fed PAGR1 mKO mice (Figure S5H, Supporting Information). Additionally, the expression levels of macrophage and profibrotic genes were markedly lower, indicating a significant reduction in HFD‐induced inflammation and fibrosis (Figure S5I, Supporting Information). These findings collectively highlight the consistent phenotypes between PAGR1 mKO and PAGR1*^f/f/Hsa‐Cre^* mice, providing strong evidence that muscle‐specific deletion of PAGR1 protects against HFD‐induced hepatic steatosis and adipose tissue inflammation.

### 2.5 Muscle PAGR1 Deficiency Enhances the Glucose Utilization

**Figure 4:**
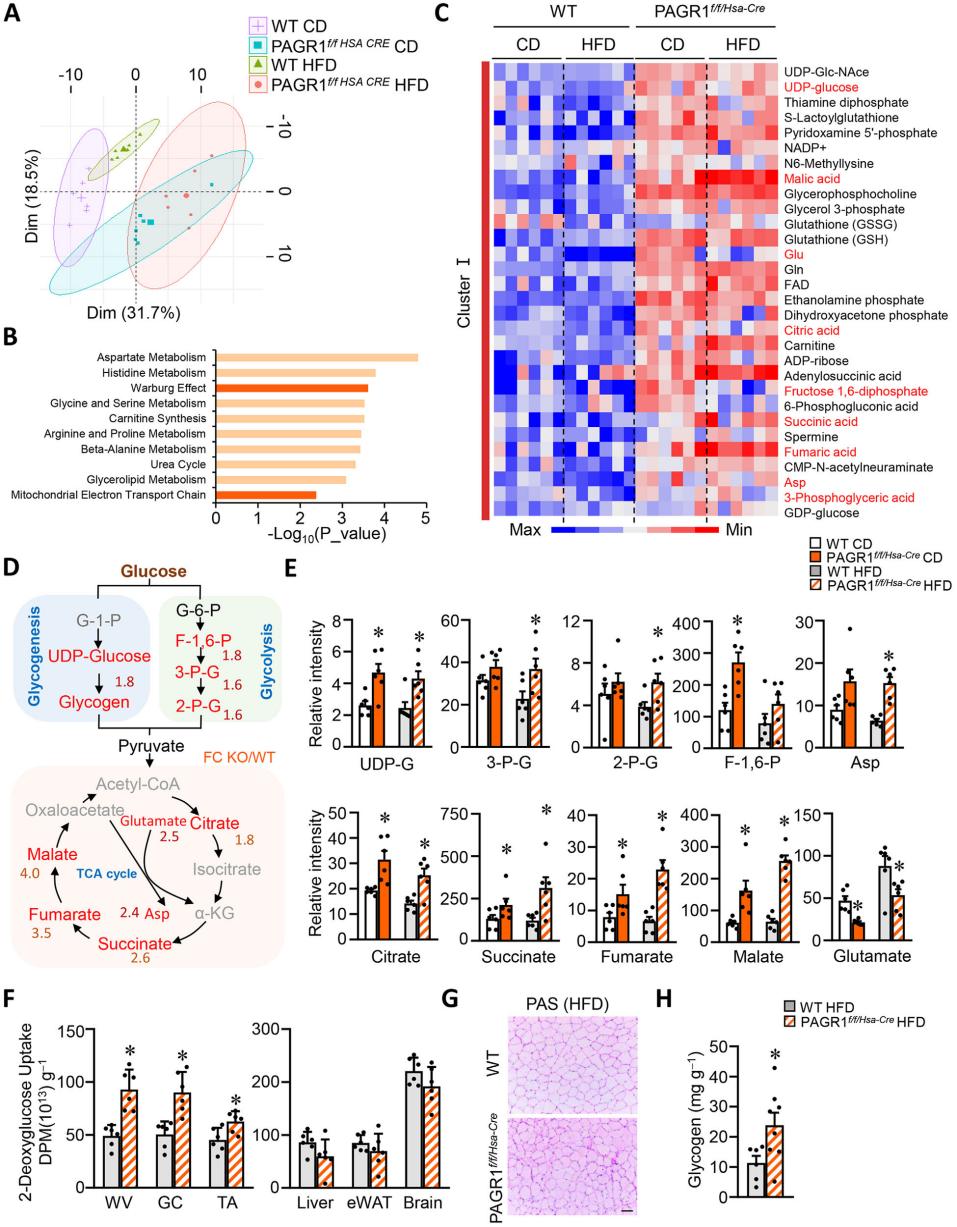
PAGR1 ablation enhances muscle glucose utilization. A) Principal component analysis (PCA) of metabolite profiles in WV muscle from four groups: PAGR1*^f/f/Hsa‐Cre^* mice and WT controls on a chow diet or HFD. Capillary electrophoresis–mass spectrometry (CE–MS)‐based metabolite analysis was performed on WV muscles from the indicated groups. *n* = 6 per group. B) Enriched metabolic pathways identified through pathway analysis of significantly altered metabolite clusters. C) Heatmap showing significantly altered metabolites in WV muscles. *p* < 0.05, paired two‐sample Wilcoxon test. Red indicates an increase, and blue indicates a decrease in metabolite levels. *n* = 6 mice per group. D) Diagram illustrating glucose metabolism and the TCA cycle. Differentially altered metabolites are mapped according to the KEGG metabolic pathway. Metabolites with significant changes are shown in red, nonsignificant metabolites in black, and undetected metabolites in gray. Red numbers indicate fold change in the HFD‐fed condition. E) Normalized intensity of metabolites involved in glycogenesis, glycolysis, and the TCA cycle. *n* = 6 mice per group. F) Measurement of d‐[^14^C]‐glucose uptake in various tissues, including WV, GC, and TA muscles, liver, eWAT, and brain, from PAGR1*^f/f/Hsa‐Cre^* mice and WT controls. *n* = 6 mice per group. G) PAS staining of TA muscle sections from PAGR1*^f/f/Hsa‐Cre^* mice and WT controls. The scale bar represents 50 µm. *n* = 5 mice per group. H) Glycogen content in TA muscle from PAGR1*^f/f/Hsa‐Cre^* mice and WT controls. *n* = 6–8 mice per group. All data are presented as the mean ± SEM. *p < 0.05 versus the corresponding WT controls, as determined by two‐tailed unpaired Student's *t*‐test (F, H) and Mann–Whitney test (E).

On the basis of the above data, we hypothesized that PAGR1 deficiency may promote beneficial metabolic reprogramming within skeletal muscle. To test this, we performed comprehensive metabolomic profiling of skeletal muscle from PAGR1*^f/f/ Hsa‐ Cre^* mice and their WT littermate controls under CD and HFD conditions. A broad panel of metabolites, including Krebs cycle intermediates, amino acids, acyl‐carnitines, and free fatty acids (FFAs), was analyzed using capillary electrophoresis–mass spectrometry (CE–MS) and liquid chromatography‐mass spectrometry (LC–MS). More than 200 metabolites were measured (**Figure** **4**A–C). Principal component analysis (PCA) revealed a distinct shift in the global metabolite profile of PAGR1‐deficient muscle under HFD conditions, indicating significant metabolic alterations driven by PAGR1 ablation (Figure 4A). Metabolite set enrichment analysis identified significant enrichment in pathways related to the Warburg effect and the mitochondrial electron transport chain (Figure 4B). The differentially expressed metabolites were categorized into four clusters (Cluster I: 30 metabolites, Cluster II: 44 metabolites, Cluster III: 10 metabolites, and Cluster IV: 14 metabolites) (Figure 4C and Figure S6A,B (Supporting Information)). Cluster I showed prominently upregulated metabolites in PAGR1‐deficient muscles under both chow and high‐fat diet conditions, suggesting that these were central to PAGR1‐mediated metabolic reprogramming (Figure 4C). Cluster I includes key intermediates in glucose metabolism, particularly glycolytic intermediates and TCA cycle metabolites. Specifically, muscles lacking PAGR1 exhibited higher levels of glycolytic intermediates (e.g., fructose 1,6‐bisphosphate and 3‐phosphoglyceric acid), glycogenesis intermediates (e.g., UDP‐glucose), and TCA cycle metabolites (e.g., citric acid, fumaric acid, succinic acid, and malic acid) compared to WT muscles (Figure 4D). The coordinated elevation of these metabolites suggests that PAGR1 deficiency enhances glucose catabolism and mitochondrial oxidative metabolism in skeletal muscle, independent of dietary fat content. This indicates a key role for PAGR1 in the regulation of muscle glucose metabolism. Cluster II, influenced by HFD feeding and PAGR1 deficiency, was enriched in glycine and serine metabolism, while Cluster III suggested that PAGR1 deficiency reduced alanine metabolism (Figure S6B, Supporting Information). Further LC–MS‐based lipidomic analysis showed that levels of FFA acyl‐carnitines, products of mitochondrial fatty acid oxidation, were comparable between PAGR1‐deficient and WT muscles under HFD conditions (Figure S6C,D, Supporting Information).

The muscle's ability to import and store glucose is crucial for systemic metabolic regulation. To assess this, we evaluated glucose uptake using [^14^C]‐labeled glucose. PAGR1*^f/f/Hsa‐Cre^* mice demonstrated significantly increased glucose uptake in several muscle groups, including white vastus (WV), GC, and tibialis anterior (TA) muscles, while glucose uptake in the liver, WAT, and brain remained similar between PAGR1*^f/f/Hsa‐Cre^* mice and WT controls (Figure 4F). Periodic acid Schiff (PAS) staining of skeletal muscle sections further supported increased glycogen levels in HFD‐fed PAGR1*^f/f/Hsa‐Cre^* mice (Figure 4G). Biochemical assays confirmed that glycogen levels were elevated in the muscles of both PAGR1 mKO and PAGR1*^f/f/Hsa‐Cre^* mice (Figure 4H). These findings indicate that the loss of PAGR1 enhances muscle glucose uptake and storage. Overall, this study suggests that PAGR1 deletion in skeletal muscle enhances glucose metabolism by promoting increased glucose uptake, disposal, and glycogen storage. This insulin‐sensitizing effect improves whole‐body insulin response and protects the liver from developing hepatic steatosis.

### 2.6 PAGR1 Coordinates Gene Programs Regulating PI3K–AKT Signaling Pathway and Glucose Metabolism

To uncover the mechanisms by which muscle PAGR1 depletion enhances glucose metabolism, we conducted a comprehensive transcriptomic analysis of skeletal muscle under conditions of PAGR1 deletion, HFD feeding, or their combination. A total of 2662 differentially expressed genes were identified using a cutoff of 1.5‐fold change and *p* < 0.05. In PAGR1*^f/f/Hsa‐Cre^* mice, we observed 312 genes significantly regulated by HFD, while 2223 and 2514 genes were affected by PAGR1 deletion under CD and HFD, respectively (Figure S7A, Supporting Information). The differentially expressed genes were categorized into four clusters: Cluster I (312 genes), Cluster II (183 genes), Cluster III (1090 genes), and Cluster IV (1077 genes). Clusters III and IV represented the most prominently PAGR1‐regulated genes, which were altered under both CD and HFD conditions (Figure S7A, Supporting Information). Cluster I consisted of HFD‐induced genes that were suppressed by PAGR1 deficiency, whereas Cluster II included genes downregulated by PAGR1 deletion during CD (Figure S7A, Supporting Information). KEGG pathway analysis of the different clusters revealed interesting findings. Cluster I, containing HFD‐induced genes suppressed by PAGR1 ablation, was enriched for the fatty acid metabolism pathway (Figure S7B, Supporting Information). These results aligned with previous lipidomic analyses, which showed comparable levels of FFA acyl‐carnitines between PAGR1‐deficient muscles and WT controls, suggesting that improved metabolic homeostasis in PAGR1*^f/f/Hsa‐Cre^* mice under HFD conditions is unlikely to be due to enhanced fatty acid metabolism. Clusters III and IV were enriched for the PI3K–AKT signaling pathway and insulin resistance, providing significant insights into the improved glucose tolerance observed in PAGR1*^f/f/Hsa‐Cre^* mice (Figure S7B,C, Supporting Information).

**Figure 5:**
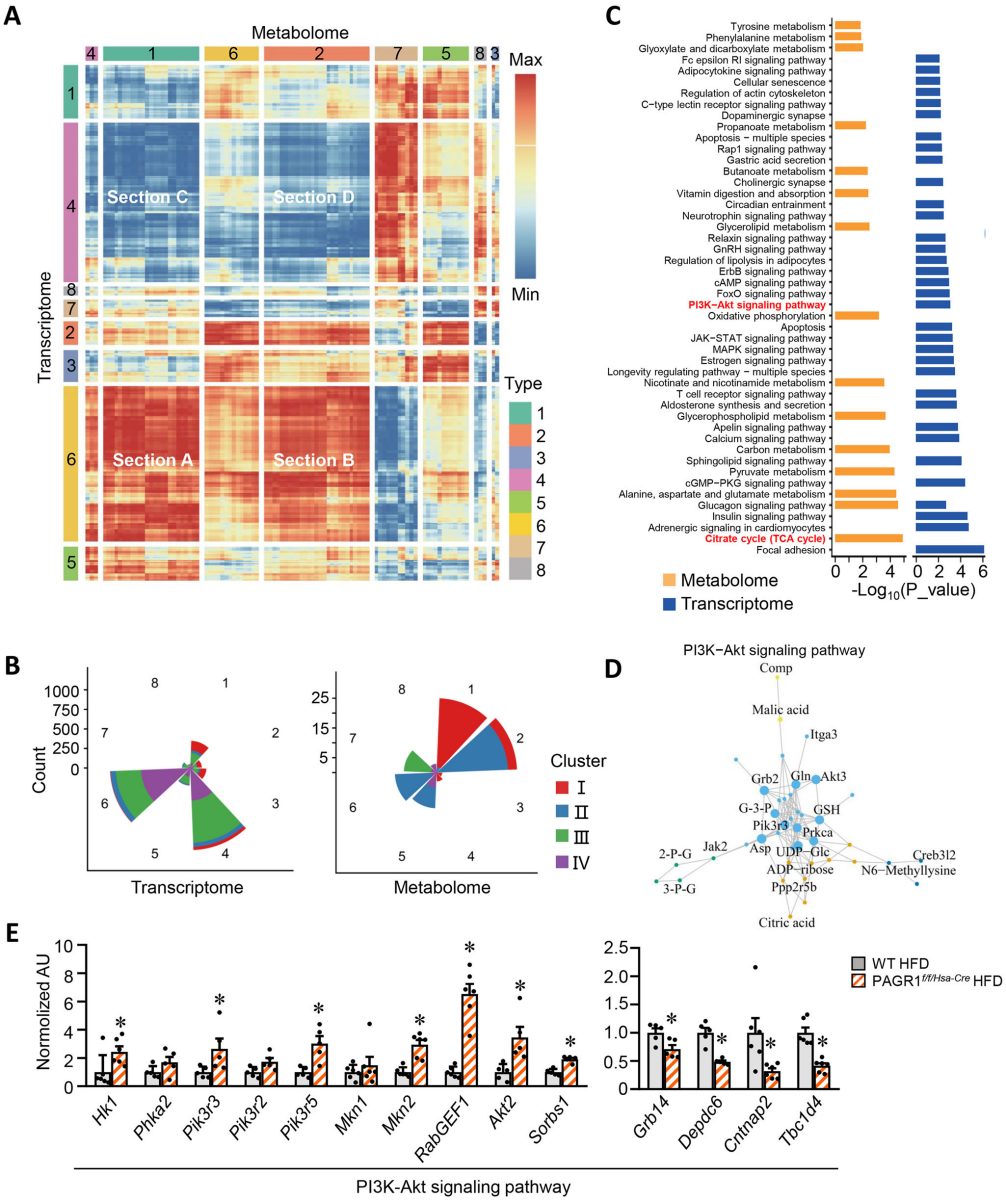
PAGR1 coordinates gene programs regulating PI3K–AKT signaling pathway and glucose metabolism. A) Integrative analysis of metabolism and transcriptomics. Heatmap depicting the clustering of metabolites (columns) and genes (rows) based on their correlations. Each cell represents the Pearson correlation coefficient between a metabolite and a gene. Metabolites and genes in Section A exhibit particularly high correlations. Columns and rows are clustered into eight types, annotated by the colored bars on the top and left sides. B) Radar plot showing the distribution of genes (top) and metabolites (bottom) within each newly defined type. Colors represent the original clusters based on transcriptomics (top) or metabolomics (bottom). C) KEGG pathway enrichment analysis for metabolites and genes within the high‐correlation Section A. Bar lengths indicate −log10(*p* value), with *p* values calculated using the enricher function of the R package clusterProfiler. Pathway enrichment in metabolites is shown in yellow, while pathway enrichment in genes is shown in blue. D) Metabolite–gene correlation network for the PI3K–AKT signaling pathway within Section A. Only metabolites with a Pearson correlation coefficient > 0.97 with genes involved in the PI3K–AKT signaling pathway are shown. The complete network is available in Figure S6C (Supporting Information). E) qRT‐PCR analysis of genes related to the PI3K–AKT signaling pathway and fatty acid metabolism in GC muscles from HFD‐fed PAGR1*^f/f/Hsa‐Cre^* mice and WT controls. *n* = 5–6 mice per group. All data are presented as the mean ± SEM. *p < 0.05 versus the corresponding WT controls, determined by two‐tailed unpaired Mann–Whitney test (E).

To further investigate the link between gene expression and metabolic state, we conducted an integrative analysis of metabolomics and transcriptomics. Spearman correlation coefficients were calculated between all metabolites and genes, and both were clustered into different types based on the correlation matrix (**Figure** **5**A). A radar plot illustrated the number of genes and metabolites in each cluster (Figure 5B). Metabolomics Cluster I, enriched in Type 1, was highly correlated with transcriptomics Type 6, forming what we designated as Section A. KEGG pathway analysis of Section A revealed significant enrichment for the PI3K–AKT signaling pathway and the TCA cycle (Figure 5C,D). Further analysis demonstrated that PAGR1 deficiency led to increased expression of PI3K–AKT signaling pathway genes (e.g., *Hk1*, *Phka2*, *Pik3r3*, *Pik3r5*, *Mkn2*, *Akt2*, *Sorbs1*) and decreased expression of genes that negatively regulate this pathway (e.g., *Grb14*, *Cntnap2*, *Depdc6*, *Tbc1d4*) (Figure 5E). Notably, the expression levels of *Appl1* and *Appl2* were not significantly altered in PAGR1‐deficient skeletal muscle (Figure S7D,E, Supporting Information). Whereas the mRNA expression levels of *Tbc1d1*, *Rab10*, and *Rab14* were not altered, *Rab5* mRNA expression was significantly increased in PAGR1*^f/f/Hsa‐Cre^* muscle (Figure S7D,E, Supporting Information). These results indicate that the PI3K–AKT signaling pathway is activated in PAGR1‐deficient muscle, contributing to enhanced glucose metabolism.

### 2.7 *Tbc1d4* Is a Direct PAGR1 Target in Skeletal Muscle and Regulates GLUT4 Translocation to the Cell Membrane

**Figure 6:**
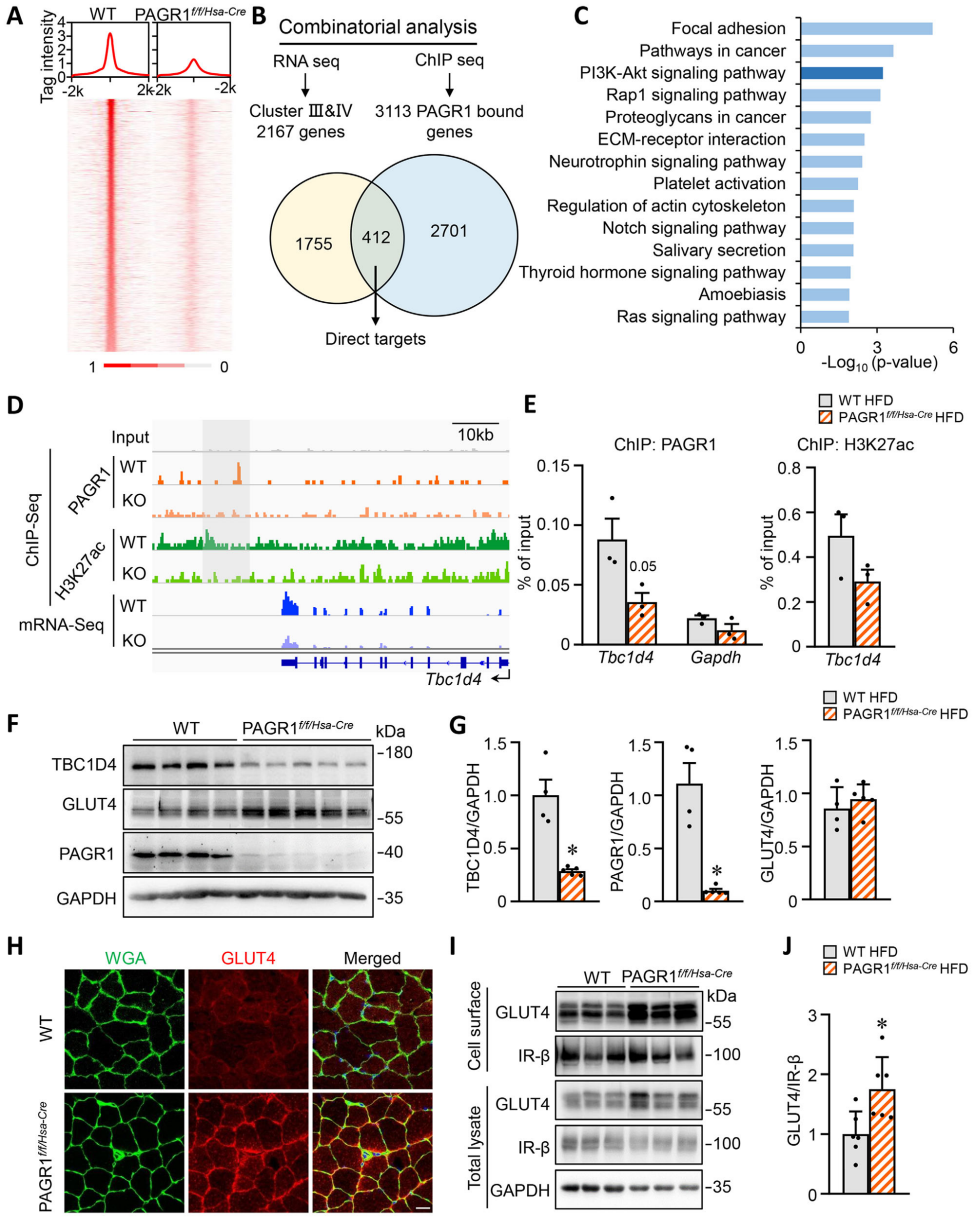
*Tbc1d4* is a direct PAGR1 target in skeletal muscle and regulates GLUT4 translocation to the cell membrane. A) Top: signal intensity plot of PAGR1 ChIP‐Seq signal in WT and PAGR1*^f/f/Hsa‐Cre^* chromatin. Bottom: heatmap of PAGR1 ChIP‐Seq signals centered around PAGR1 binding sites. B) Integration of PAGR1 ChIP‐Seq data with mRNA‐Seq data from muscle following PAGR1 deletion, identifying a set of genes directly regulated by PAGR1. C) GO enrichment analysis of PAGR1 direct target genes, showing the top 14 enriched terms. D) PAGR1‐dependent binding at the *Tbc1d4* gene locus. Top: ChIP‐Seq binding profiles for PAGR1 and histone modifications (H3K27ac) in WT and PAGR1*^f/f/Hsa‐Cre^* muscle. Bottom: mRNA‐Seq data showing correlation with ChIP‐Seq results. Gray boxes highlight high‐confidence PAGR1‐binding regions in the *Tbc1d4* gene. Input: genomic DNA from skeletal muscle. For PAGR1 ChIP‐seq and H3K27ac ChIP‐Seq, gastrocnemius muscles from three animals were pooled per group. E) ChIP assays for PAGR1 and H3K27ac recruitment at the *Tbc1d4* peak in WT and PAGR1*^f/f/Hsa‐Cre^* muscles. *n* = 3 mice per group. F) Representative Western blot analysis of TBC1D4 protein levels in GC muscle from WT and PAGR1*^f/f/Hsa‐Cre^* mice. *n* = 4–6 mice per group. G) Quantification of TBC1D4/GAPDH signal ratios, normalized to 1.0 for WT under HFD and shown below the corresponding bands. *n* = 4–5 mice per group. H) Representative images of GLUT4 immunostaining in GC muscle fibers from WT and PAGR1*^f/f/Hsa‐Cre^* mice. WGA (wheat germ agglutinin) is used as a membrane marker. Scale bar represents 50 µm. *n* = 4 mice per group. I) Cell surface and total GLUT4 levels in WV muscle from WT and PAGR1*^f/f/Hsa‐Cre^* mice. J) Quantification of GLUT4/IR‐β signal ratios, normalized to 1.0 for WT and shown below the corresponding bands. *n* = 6 mice per group. All data are presented as the mean ± SEM. *p < 0.05 versus the corresponding WT controls, determined by two‐tailed unpaired Student's *t*‐test (E) and Mann–Whitney test (G, J).

To investigate the genome‐wide PAGR1 binding profile in WT mouse muscle chromatin, we performed ChIP‐Seq analysis. As a control, ChIP‐Seq was also conducted in PAGR1*^f/f/Hsa‐Cre^* mice muscles, revealing 4594 high‐confidence PAGR1 genomic binding regions after filtering out nonspecific signals observed in PAGR1‐deficient muscle (Figure S8C, Supporting Information). The PAGR1‐binding sites in skeletal muscle were predominantly located in intergenic, intronic, and promoter regions (**Figure** **6**A). Integrating the PAGR1 cistrome data with global mRNA changes following muscle PAGR1 deletion showed that ≈412 PAGR1‐regulated genes were directly bound by PAGR1 (identified as genes nearest to the PAGR1‐binding peaks) (Figure 6B). GO analysis revealed that these direct target genes were significantly enriched in the PI3K–AKT signaling pathway, consistent with the transcriptomic profile observed in PAGR1*^f/f/Hsa‐Cre^* mice muscle (Figure 6C). This strong correlation between cistromic and transcriptomic data underscores the direct role of PAGR1 in regulating metabolic gene programs in muscle. Visualization of PAGR1 binding and H3K27ac signals at the *Tbc1d4* locus aligned with RNA‐Seq and gene expression results, indicating that PAGR1 positively regulates *Tbc1d4* transcription (Figure 6D). ChIP‐q‐polymerase chain reaction (PCR) assays on PAGR1‐deficient muscle confirmed reduced PAGR1 binding and decreased H3K27ac levels at the *Tbc1d4* gene, supporting direct transcriptional regulation by PAGR1 (Figure 6E). Western blot analysis further demonstrated that the protein level of TBC1D4 was reduced in the muscle of PAGR1*^f/f/Hsa‐Cre^* mice, consistent with gene expression data (Figure 6F,G). Interestingly, genome‐wide H3K4me1 profiles remained largely unchanged upon PAGR1 deletion, and we observed no significant alterations in H3K4me1 enrichment at the *Tbc1d4* locus (Figure S8D,E, Supporting Information). Total H3K27ac/H3 levels were not significantly altered in PAGR1*^f/f/Hsa‐Cre^* skeletal muscle (Figure S8F, Supporting Information), suggesting that the local enhancer activity at *Tbc1d4* may be impaired in the absence of PAGR1. Notably, HDAC inhibitor sodium butyrate treatment effectively elevated global H3K27ac levels, but it failed to rescue TBC1D4 protein expression (Figure S8G, Supporting Information). We also performed an additional experiment using an AKT‐specific inhibitor (AKTi‐1/2), despite effective suppression of AKT activity, the reduced TBC1D4 protein levels in PAGR1‐deficient muscle remained unchanged (Figure S9A, Supporting Information).

In the context of the PI3K–AKT signaling pathway, TBC1D4 acts as a RabGAP that inhibits GLUT4 translocation and is regulated by AKT‐mediated phosphorylation, which is critical for GLUT4 movement and glucose uptake in skeletal muscle. We found that GLUT4 translocation to the cell surface was markedly increased in PAGR1‐deficient muscle during HFD feeding, as shown by GLUT4 immunofluorescence of cell surface proteins (Figure 6H), while the expression of GLUT1 protein levels was not different in PAGR1‐deficient muscle compared to controls (Figure S9B, Supporting Information). This increased GLUT4 translocation was further confirmed by an immunoblot assay, demonstrating higher levels of GLUT4 on the plasma membrane in PAGR1*^f/f/Hsa‐Cre^* muscle during HFD feeding (Figure 6I). Moreover, insulin injection significantly increased GLUT4 translocation to the plasma membrane in PAGR1*^f/f/Hsa‐Cre^* muscle compared to WT controls (Figure S9C, Supporting Information). Taken together, these findings suggest that PAGR1 directly regulates *Tbc1d4* transcription to control GLUT4 translocation to the cell membrane, enhancing glucose uptake and utilization in skeletal muscle.

**Figure 7:**
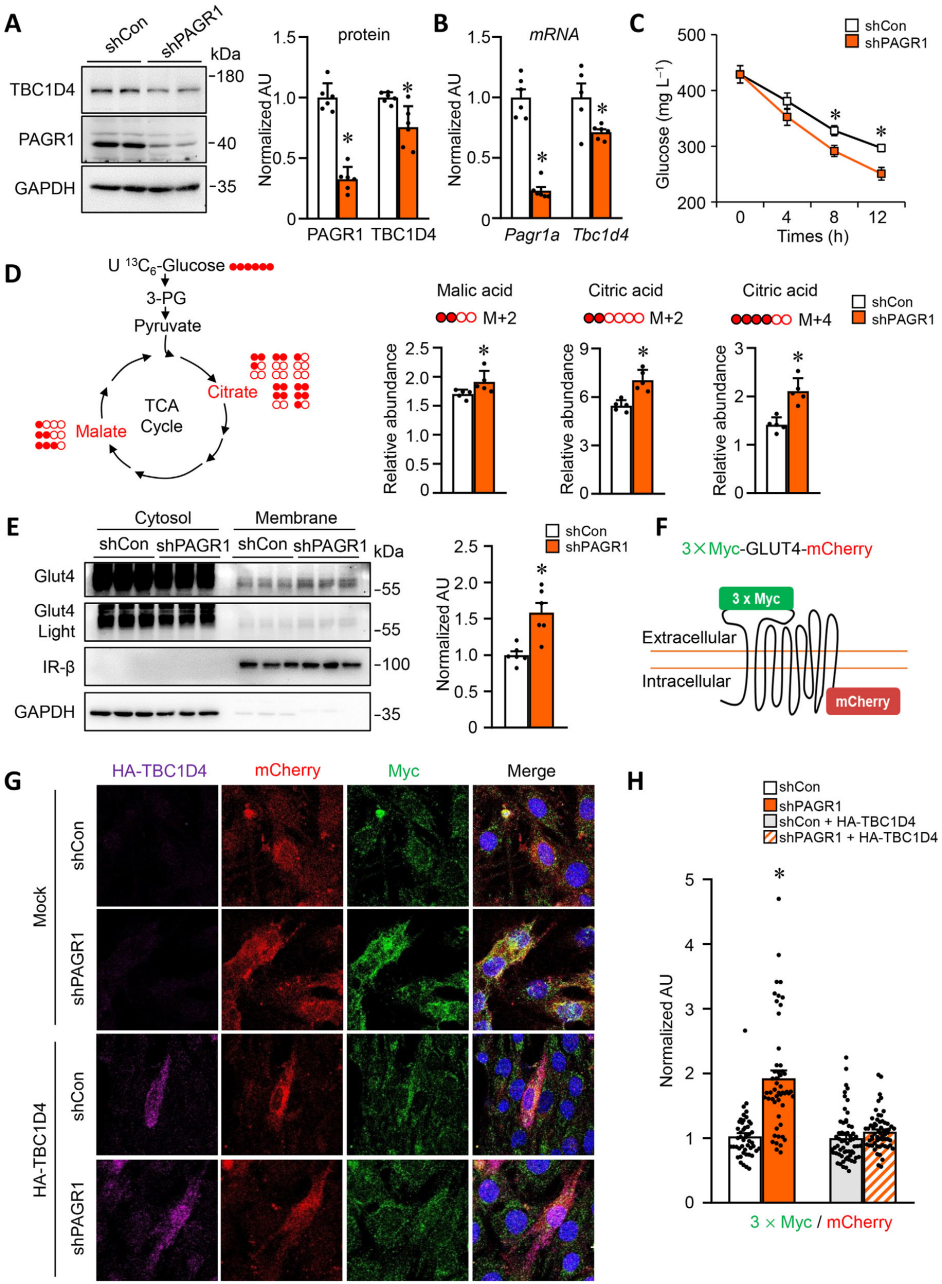
PAGR1 modulates glucose uptake by regulating TBC1D4 expression and GLUT4 translocation to the cell surface in muscle cells. A) Left: representative Western blot analysis of PAGR1 and TBC1D4 protein levels in C2C12 myotubes transduced with shControl (shCon) or shPAGR1. Right: quantification of PAGR1/GAPDH and TBC1D4/GAPDH signal ratios, normalized to 1.0 for the control group. *n* = 5 per group. B) qRT‐PCR analysis of *Pagr1a* and *Tbc1d4* mRNA expression in C2C12 myotubes with shCon and shPAGR1. *n* = 5 per group. C) Glucose consumption in the culture medium of C2C12 myotubes containing 25 mm glucose, measured at different time points. *n* = 6 per group. D) Left: schematic representation of ^13^C‐labeled glucose tracing in shCon and shPAGR1 C2C12 myotubes. Right: kinetic accumulation of uniformly labeled citrate (M+2, M+4) and malate (M+2) in shCon and shPAGR1 C2C12 myotubes 30 min after the addition of 25 mm ^13^C‐glucose to the medium. *n* = 5 per group. E) Left: analysis of cell surface and cytosolic GLUT4 levels in shCon and shPAGR1 C2C12 myotubes. Right: quantitative data of cell surface GLUT4 levels. *n* = 6 per group. F) Schematic illustration of a GLUT4 fusion protein used for detecting GLUT4 translocation and plasma membrane (PM) surface exposure. G) Representative confocal microscopy images showing GLUT4 surface exposure in shCon and shPAGR1 C2C12 cells after 16 h of serum starvation. Scale bar represents 20 µm. H) Quantification of the ratio of surface to total GLUT4, assessed by detecting surface GLUT4 using anti‐Myc fluorescence labeling and total GLUT4 with mCherry fluorescence in nonpermeabilized cells. Data shown are representative confocal images of ≈30–60 cells per group from three independent experiments. All data are presented as the mean ± SEM. *p < 0.05 versus the corresponding WT controls, determined by two‐tailed unpaired Student's *t*‐test (A, B, H), Mann–Whitney test (D, E), and two‐way ANOVA (C) followed by Fisher's LSD post‐hoc test.

To further investigate these findings in a cell‐autonomous context, we conducted a comparative metabolomic analysis in C2C12 myotubes transduced with lentiviruses expressing either scramble or PAGR1 shRNA. Consistent with in vivo observations, PAGR1 knockdown led to a reduction in both *Tbc1d4* mRNA and TBC1D4 protein levels (**Figure** **7**A,B). We assessed glucose consumption by measuring glucose levels in the culture medium and found that PAGR1 knockdown in C2C12 myotubes enhanced glucose uptake, as indicated by decreased glucose concentrations in the medium (Figure 7C). To confirm the enhanced glucose utilization in PAGR1‐deficient muscle cells, we examined glycolytic and TCA cycle flux using isotopic [^13^C]‐glucose tracing experiments (Figure 7D). PAGR1 knockdown myotubes exhibited increased incorporation of ^13^C into TCA cycle intermediates, such as citrate (M+2 and M+4) and malate (M+2), indicating enhanced glucose utilization and flux through the TCA cycle (Figure 7D). These results align with the steady‐state metabolic data observed in PAGR1*^f/f/Hsa‐Cre^* muscle, suggesting that loss of PAGR1 promotes glucose utilization in myotubes in a cell‐autonomous manner.

Consistent with in vivo findings, total GLUT4 protein levels is unchanged in PAGR1 knockdown myotubes (Figure 7E). We next evaluated GLUT4 translocation in C2C12 myotubes. Supporting the increased glucose utilization, GLUT4 translocation to the cell surface was elevated in PAGR1 knockdown myotubes. To visualize GLUT4 translocation, we engineered a Myc–GLUT4–mCherry fusion protein, with the 3 × Myc epitope inserted into the first exofacial loop of the GLUT4 N‐terminus and mCherry fused at the C‐terminus, allowing detection of plasma membrane (PM) insertion (via Myc under nonpermeabilized conditions) and total GLUT4 content (via mCherry) (Figure 7F), similar to previously reported constructs.^[^ ^15^ ^]^ Following 16 h of serum starvation, PAGR1 silencing increased GLUT4 localization at the cell surface compared to control myotubes, while overexpression of TBC1D4 blocked this effect in PAGR1 knockdown myotubes (Figure 7F–H). Collectively, these data support the role of PAGR1 in modulating glucose uptake by regulating TBC1D4 expression and enhancing GLUT4 translocation to the cell surface in muscle cells.

## 3 Discussion

**Figure 8:**
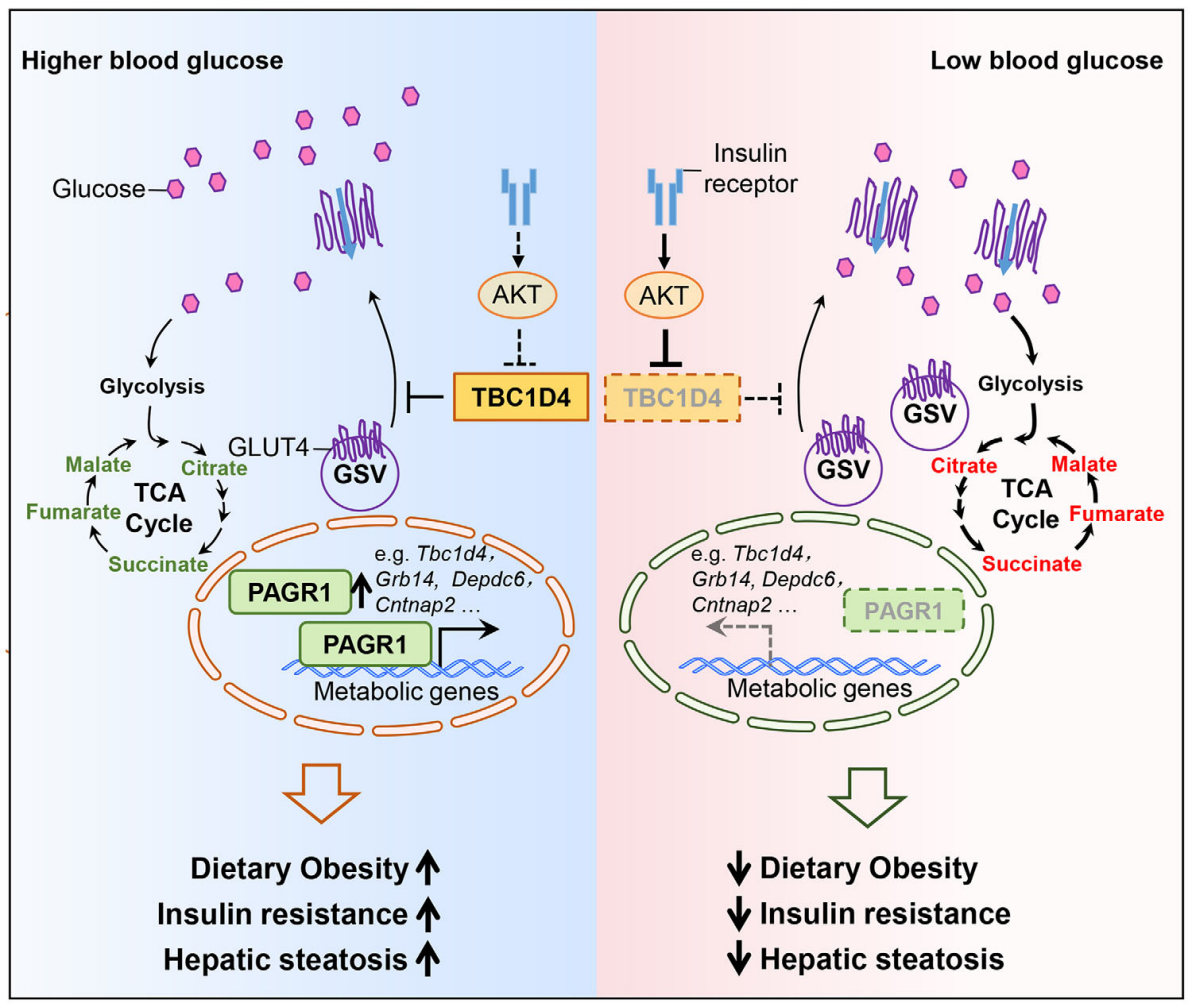
The schematic depicts the proposed model for the glucose‐responsive PAGR1‐regulated skeletal muscle gene program controls systemic glucose homeostasis and hepatic metabolism.

Glucose toxicity, characterized by chronic hyperglycemia, poses significant challenges to glucose utilization in skeletal muscle, a primary site for postprandial glucose disposal and whole‐body glycemic control. The mechanisms by which hyperglycemia disrupts skeletal muscle function and contributes to insulin resistance remain unclear. In our study, we identify PAGR1 as a glucose‐responsive regulator that governs skeletal muscle glucose metabolism and systemic metabolic homeostasis (**Figure** **8**). We found that PAGR1 expression is induced by high glucose levels, and its deficiency in muscle leads to enhanced insulin signaling, increased GLUT4 translocation, and improved glucose uptake and utilization. These findings uncover a PAGR1‐dependent gene program that supports adaptive glucose metabolism in muscle and protects against diet‐induced obesity and hepatic steatosis. Importantly, our results suggest that PAGR1 links skeletal muscle glucose handling to systemic glucose homeostasis and highlights the potential for targeting PAGR1 to counteract the detrimental effects of chronic hyperglycemia.

Epigenetic regulation is pivotal for enabling adaptive gene expression in response to metabolic and environmental stimuli, which significantly impacts metabolic health and disease. While PAGR1 has been identified as an epigenetic cofactor associated with the MLL3/MLL4 histone methyltransferase complex,^[^ ^13^ ^]^ its functions and mechanisms, particularly in metabolic contexts, have remained largely unexplored. Our study uncovered a new role for PAGR1 in linking extracellular glucose levels to a transcriptional program that affects AKT activation and glucose utilization in skeletal muscle, which likely contributes to systemic metabolic improvements, including reduced adiposity. This work establishes a previously unrecognized molecular pathway where PAGR1 responds to high glucose stimuli, facilitating TBC1D4 regulation and inhibiting GLUT4 translocation to the cell membrane, ultimately regulating muscle glucose uptake. Notably, unlike MLL4, which was shown to be highly expressed in type I muscle fibers and essential for establishing and maintaining type I muscle fibers,^[^ ^16^ ^]^ PAGR1 does not influence type I muscle fiber composition. Our results suggest that PAGR1 regulates *Tbc1d4* expression independently of its canonical association with the MLL3/MLL4 histone H3K4 methyltransferase complex. Instead, PAGR1 likely exerts context‐dependent transcriptional control in skeletal muscle through distinct, noncanonical mechanisms. While H3K27ac levels at the *Tbc1d4* locus are reduced, our findings suggest that PAGR1's regulation of *Tbc1d4* is not solely dependent on H3K27 acetylation and may involve other mechanisms, such as recruitment of transcriptional coregulators or modulation of chromatin accessibility.

While pancreatic beta cells are classically recognized for their role in blood glucose sensing and insulin secretion, emerging evidence underscores that skeletal myocytes are also capable of sensing extracellular glucose concentrations and activating glucose‐responsive signaling pathways. Adaptive mechanisms in muscle, such as the Baf60c–Deptor–AKT pathway, have been shown to promote glucose‐stimulated AKT activation and enhanced insulin action.^[^ ^17^ ^]^ We demonstrate that PAGR1 integrates glucose signals to regulate TBC1D4‐mediated GLUT4 translocation, orchestrating a glucose disposal response that significantly impacts whole‐body glucose balance. Our targeted lipidomic analysis revealed that levels of diacylglycerols and ceramides were comparable between PAGR1‐deficient and WT control muscles under HFD conditions. These data suggest that the improved insulin signaling and glucose uptake observed in PAGR1 knockout mice occur independently of reductions in these toxic lipid species. TBC1D4, a known Rab GTPase‐activating protein, plays a pivotal role in modulating GLUT4 vesicle movement to the plasma membrane, enabling efficient glucose uptake in response to insulin signaling.^[^ ^6^, ^18^ ^]^ Our findings suggest that PAGR1 primarily regulates glucose metabolism by modulating key components of the GLUT4‐specific trafficking machinery. The increased *Rab5* expression in PAGR1*^f/f/Hsa‐Cre^* muscle also suggests enhanced endosomal trafficking and vesicle transport activity, consistent with the observed increase in GLUT4 translocation. However, it is possible that PAGR1 also modulates other components within the AKT signaling pathway and its potential interactions with alternative mechanisms that support GLUT4 translocation and glucose utilization. Our results suggest that the regulation of TBC1D4 by PAGR1 occurs independently of AKT activation. Given that AKT is known to modulate TBC1D4 primarily through phosphorylation rather than transcriptional control, our data support a model in which PAGR1 maintains basal TBC1D4 expression at the transcriptional level, whereas AKT fine‐tunes TBC1D4 activity via posttranslational phosphorylation. At any rate, this newly identified glucose‐induced, PAGR1‐regulated pathway in skeletal muscle offers new avenues for potential therapeutic strategies to optimize glucose metabolism and combat metabolic disorders such as type 2 diabetes.

While our data suggest that the metabolic reprogramming in skeletal muscle‐specifically enhanced glucose disposal and improved insulin action is a primary contributor to the observed systemic metabolic improvements, including reduced adiposity, the mechanisms directly linking muscle PAGR1 deletion to reduced adiposity warrant further investigation. Muscle‐to‐other organ crosstalk is increasingly recognized as a significant factor in the regulation of systemic metabolism.^[^ ^19^ ^]^ Our study demonstrates that muscle‐specific PAGR1 deletion in PAGR1*^f/f/Hsa‐Cre^* mice prevents hepatic steatosis upon HFD feeding. This finding suggests that PAGR1 plays a role in muscle that influences liver metabolism. Specifically, we observed that PAGR1 ablation in muscle led to decreased free fatty acid uptake and de novo lipogenesis in the liver, indicating that PAGR1 acts within skeletal muscle to modulate the hepatic response to nutrient excess. In addition to the liver, our data indeed revealed notable effects of skeletal muscle‐specific PAGR1 deletion on adipose tissue remodeling. Although muscle‐derived cytokines, or myokines, are known mediators of interorgan metabolic communication,^[^ ^20^ ^]^ our findings do not implicate classic myokines such as irisin, interleukin‐6, FGF21, or FGF family members in the PAGR1‐dependent muscle‐to‐other organ crosstalk.^[^ ^20^, ^21^ ^]^ While our data do not support a role for these classical myokines, we cannot rule out the involvement of other muscle‐derived factors or metabolites in mediating muscle‐adipose tissue crosstalk. Indeed, our data suggest that the enhanced muscle fuel utilization in PAGR1*^f/f/Hsa‐Cre^* mice contributes to the observed hepatic protection. Intriguingly, we also detected elevated levels of various amino acid species in PAGR1‐deficient muscles, which may indicate increased glucose flux into the TCA cycle or heightened muscle protein turnover. Given recent studies showing that amino acid catabolism can influence muscle insulin sensitivity,^[^ ^22^ ^]^ it is plausible that altered amino acid metabolism could play a role in PAGR1's metabolic actions. This warrants further investigation to define the precise mechanisms by which muscle PAGR1 deletion influences liver and adipose tissue remodeling. Notably, a high‐throughput functional screen using *Drosophila melanogaster* was performed to evaluate candidate genes located at human body mass index‐associated GWAS loci.^[^ ^23^ ^]^ This study provides a compelling functional link between *PAGR1* and adiposity‐related traits. These findings warrant further investigation into the potential contribution of *PAGR1* genetic variation to human metabolic disease susceptibility.

In summary, our study reveals that PAGR1 functions as a crucial glucose‐responsive regulator in skeletal muscle, regulating systemic glucose homeostasis and hepatic metabolism. Muscle‐specific deletion of PAGR1 improves insulin sensitivity, glucose uptake, and protects against HFD‐induced obesity and liver steatosis by enhancing GLUT4 translocation via TBC1D4 regulation. These findings highlight PAGR1 as a potential target for therapeutic strategies aimed at mitigating glucotoxicity and combating metabolic diseases.

## 4 Experimental Section

### Mice

All animal studies were conducted in strict accordance with the institutional guidelines for the humane treatment of animals and were approved by the IACUC committees at the Model Animal Research Center of Nanjing University (Approval No. GZJ07). Male C57BL/6J wild‐type mice were from GemPharmatech Co., Ltd. (Jiangsu, China). PAGR1*^fl/fl^* mice were generated as previously described.^[^ ^14b^ ^]^ Mice were backcrossed to the C57BL/6J background for more than 6 generations. To generate mice with a muscle‐specific disruption of the PAGR1 allele, PAGR1*^fl/fl^* mice were bred with mice expressing Cre recombinase under the control of the MCK promoter (Jackson Laboratory, stock no. 006475) or the HSA promoter (Jackson Laboratory, stock no. 006139), respectively. These crosses allowed for muscle‐specific deletion of PAGR1. Male mice of the relevant genotypes and ages (3–5 months) were fed ad libitum with standard laboratory rodent chow or a high‐fat diet (60% of calories from fat, Research Diets no. D12492) as specified. For HDAC inhibition, sodium butyrate (Sigma‐Aldrich, 303410) was dissolved in 0.9% NaCl and administered to PAGR1*^f/f/Hsa‐Cre^* mice by daily intraperitoneal injection at a dose of 300 mg kg^−1^ body weight for five consecutive days. For AKT inhibition, AKTi‐1/2 (MCE, HY‐10355) was similarly prepared in 0.9% NaCl and administered to WT and PAGR1*^f/f/Hsa‐Cre^* mice at 50 mg kg^−1^ body weight for two days. The animals were kept in plastic cages under a 12 h light–dark cycle, at a temperature of 21 ± 2 °C, and provided with free access to water and pellet food. Male mice between 12 and 20 weeks of age were used for all experiments. All mice were housed in a specific pathogen‐free facility at the Nanjing University. Littermate controls were used in all experiments.

### Metabolite Extraction

Metabolite extraction of skeletal muscle tissues was conducted according to previously published methods.^[^ ^24^ ^]^ In brief, sheared skeletal muscle tissues were weighed and then 500 µL ice‐cold methanol with internal standard 1 (IS1, Human Metabolome Technologies (HMT), H3304‐1002, 1:200, used to standardize the metabolite intensity and to adjust the migration time) were added. Mixed grinding apparatus (Scientz‐48) was used for homogenization (35 Hz, 1 min) followed by addition of 500 µL chloroform and vortex for 30 s. After phase breaking using 200 µL water and centrifugation (13 000 *g*, 4 °C, 15 min), 420 µL hydrophilic layer was transferred for ultrafiltration through a 5 kDa cutoff filter (Millipore, cat. UFC3LCCNB‐HMT). Simultaneously, the quality control (QC) sample was prepared by combining the aqueous phase from each sample and then filtered. Then, samples were vacuum dried and stored at −80 °C until CE–MS analysis. For acyl‐carnitine and fatty acid analysis using LC–MS, internal standards (including Carnitine C2:0_d3, Carnitine C16:0_d3, FFA 16:0_d3, and FFA 18:0_d3) were used to improve the precision of quantitative analysis and monitor the robustness of sample preparation and the stability of instrument analysis. 300 µL ice‐cold methanol with internal standards were added to tissue samples, and then submitted to mixed grinding apparatus for homogenization (35 Hz, 1 min) followed by addition of 1 mL methyl *tert*‐butyl ether and vortex for 1 min. After phase breaking using 300 µL water and centrifugation (13 000 *g*, 4 °C, 15 min), 600 µL hydrophobic layer was collected and freeze‐dried for fatty acids analysis. At the same time, the QC sample was prepared by combining the hydrophobic layer from each sample and then vacuum dried. For acyl‐carnitine analysis, 300 µL hydrophilic layer and 300 µL hydrophobic layer were freeze‐dried. QC sample was also prepared by combining the aqueous phase and then vacuum dried to evaluate the analytical quality.

### ^13^C6‐Glucose Flux

For metabolic flux analysis, [U‐^13^C_6_] glucose (from Cambridge Isotope Laboratories) was added to C2C12 myotubes for the indicated times at a final concentration of 25 mm following 30 min of starvation. Cells were washed and then quenched in liquid nitrogen and stored at −80 °C until MS analysis was performed.

### Metabolomics Analyses

CE–MS‐based metabolomics and metabolic flux analysis were conducted on CE (G7100A, Agilent) coupled to the time of flight (TOF) mass spectrometry (G6224A, Agilent). The fused silica capillary (50 µm i.d. × 80 cm, HMT, Japan) was used for sample separation. Two analysis modes (including the cation‐positive mode and the anion‐negative mode) were performed in this experiment. Detailed CE–MS methods were performed as previously described.^[^ ^25^ ^]^ A preanalyzed metabolite standard library by HMT was used for qualitative analysis of metabolites and peak extraction and identification were carried out with Quantitative Analysis Software (Agilent).

For the intensity of metabolites within TCA cycle, internal standards (IS1) were used for migration time correction for metabolites. After peak information with matched migration time, *m*/*z* value, and peak area were exported, normalization to the area of internal standards and the weight of tissue for each sample was performed to get relative intensity of metabolites within TCA cycle. For metabolic flux analysis of TCA cycle intermediates, CE–MS‐based metabolic flux analysis was also conducted. After peak extraction, the relative abundance of native M+0 and isotopologues M+*i* for identified TCA intermediates were obtained through normalizing by total peak area of whole metabolites from a cell sample.

LC–MS analysis was performed by an ACQUITY UPLC system (Waters) coupled with a tripleTOF 5600 plus mass spectrometer (AB SCIEX). The C8 AQUITY column (2.1 mm × 50 mm × 1.7 µm) was used for acyl‐carnitines separation in positive ion mode. Development and validation of this rapid method was described before.^[^ ^26^ ^]^ And C8 AQUITY column (2.1 mm × 100 mm × 1.7 µm) was used for fatty acid separation in negative ion mode and the detailed mobile phases and gradient elution were described before.^[^ ^27^ ^]^ All samples were randomized with respect to run order to avoid batch effects. Additionally, the QC samples were identically inserted into the analytical sequence to monitor the reproducibility of the analytical method. Acyl‐carnitine and fatty acid identification was based on exact mass, retention time, and MS/MS pattern. Peakview workstation (AB SCIEX) was used to check MS/MS information of metabolites and Multiquant (AB SCIEX) was used to obtain the area of identified metabolites. The applied database search engines were HMDB (http://www.hmdb.ca/), Metlin (https://metlin.scripps.edu), and LIPID MAPS (http://www.lipidmaps.org/).

Before statistical analysis, normalization to the area of internal standards and the weight of tissue for each sample was performed. For cell's metabolomics analysis, the quantity of metabolites was normalized by total peak area of whole metabolites from a cell sample. For statistical analysis, all metabolomic studies were analyzed by Student's *t* test (2‐tailed) or one‐way ANOVA coupled to a Fisher's LSD post‐hoc test when more than two groups were compared, setting *p* < 0.05 as the significant difference levels. *K*‐means cluster analysis (*K* = 4) was performed with the Cluster 3.0 for the normalized metabolite data (*p* < 0.05) from CE–MS. Metabolite‐associated pathways were analyzed using MetaboAnalyst 5.0 (Xia Lab at the McGill University, Montreal, Canada; metaboanalyst.ca). PCA and heatmap analyses were generated by using R software, version 4.0.3 with the factoextra/FactoMineR/ggpubr and gplots packages, respectively.

### [^14^C]2‐Deoxyglucose (DG) Uptake In Vivo

To assess tissue glucose uptake in vivo, the uptake of DG was measured with slight modifications to the described procedure.^[^ ^28^ ^]^ Five months old male mice were intraperitoneally injected with DG (50 µCi kg^−1^, diluted with 0.2 g kg^−1^ d‐glucose) and were killed 60 min later. Different tissues were quickly removed, weighed, and digested in 1 n NaOH (1 mL per 100 mg of tissue, wet weight) at 60 °C for 2 h. The extracts were neutralized by the addition of 2 n HCl (0.5 mL per 1 mL of 1 n NaOH), centrifuged, and aliquots of the supernatant were measured after mixing with the scintillation cocktail medium Ultima Gold XR (Perkin, US) in a Tri‐Carb 4910TR Liquid Scintillation Analyzer (Perkin, US). Glucose uptake was normalized to the wet weight of the tissue. Radioactivity was quantified using disintegrations per minute per gram of tissue as an indicator of glucose uptake capacity.

### Glucose and Insulin Tolerance Testing

The mice were fasted overnight (for GTT) or for 4 h (for ITT) prior to the studies according to previously published methods.^[^ ^29^ ^]^ For GTT studies, 1.5 g kg^−1^ of d‐glucose was administered to the mice via intraperitoneal injection. For ITT, human regular insulin (Sigma‐Aldrich) was injected intraperitoneally at a dose of 1 U kg^−1^ body weight. Blood glucose levels were measured at 0, 15, 30, 60, 90, and 120 min after the glucose or insulin challenge using a OneTouch ultramini glucose meter (OneTouch). The area under the curve (AUC) was calculated as the difference between baseline glucose levels and the deflection caused by the glucose or insulin challenge. The total AUC was calculated using the trapezoidal rule. To assess insulin signaling, mice were injected intraperitoneally with insulin at a dose of 2 U kg^−1^ body weight.

### Metabolic Measurements In Vivo

The mice were individually housed in metabolic cages with free access to food and water and a 12 h light/dark cycle using the CLAMS system (Columbus Instruments). Before the recordings, the mice were acclimated in the metabolic cage for one day. The CLAMS system was used to simultaneously assess food intake, energy expenditure, physical activity, as well as VO_2_ and VCO_2_ levels according to the manufacturer's instructions.

### Blood and Tissue Chemistry

After a 12 weeks high‐fat diet feeding, mice were fasted overnight (16 h) beginning at 5 p.m. Blood samples were collected for glucose measurements. Liver tissue (50 mg) was homogenized, and centrifuged supernatants were harvested. The TG levels were determined with the Free Glycerol Reagent (F6428, Sigma‐Aldrich) using glycerol (G7793, Sigma‐Aldrich) as standard for calculation. Serum ALT levels were determined according to the manufacturer's instructions (B01, Neusoft Whitman Biotech). Glycogen concentration in tibialis anterior muscle was measured after acid hydrolysis as previously described.^[^ ^30^ ^]^ In brief, muscle homogenates were heated (100 °C) for 2 h in 2 mol L^−1^ HCl. Glucosyl units were determined in a fraction of the supernatant (298‐65701, Wako) and normalized to muscle weight.

### Histological Analyses

Mouse muscle tissues were snap‐frozen in isopentane precooled in liquid nitrogen. To perform immunofluorescence staining, muscle fibers were labeled with antibodies specific for MHC1 (BA‐D5), MHC2b (BF‐F3) (MHC1 in green and MHC2 in red), and GLUT4 (green). For glycogen accumulation detection, the TA muscle was collected, embedded in Tissue‐Tek O.C.T. Compound (Leica), and snap‐frozen using precooled isopentane. Sections were stained with PAS. Mice livers were embedded in Tissue‐Tek OCT cryostat molds (Leica) and frozen at −80 °C. 10 µm thick sections were generated in a cryostat, and the tissue sections were stained with 0.5% oil red O and counterstained with hematoxylin and eosin (Sigma‐Aldrich) according to the standard protocol. Adipose tissues were fixed in 4% formaldehyde overnight at 4 °C immediately after sacrifice, embedded in paraffin, and cut into 10 µm sections on slides using a Leica RM2016 microtome. The sections were stained with hematoxylin and eosin.

### RNA‐Seq Studies

Transcriptomics analyses were performed using RNA‐sequencing as previously described.^[^ ^31^ ^]^ Total RNA was isolated from the gastrocnemius muscle or liver of PAGR1*^f/f/Hsa‐Cre^* mice and WT control mice fed with either CD or HFD using RNAiso Plus (Takara Bio). The experiments with 2 independent pools per group were performed. RNA‐Seq using Illumina HiSeq 4000 was performed by the Beijing Novogene Bioinformatics Technology Co. Two independent samples per group were analyzed. Paired‐end 150 nt reads were obtained from the same sequencing lane. The sequencing reads were then aligned to the UCSC mm10 genome assembly using TopHat, version 2.0.14, with the default parameters. Fragments per kb of exon per million mapped reads were calculated using Cufflinks, version 2.2.1. The criteria for a regulated gene was a fold change greater than 1.5 (either direction) and a significant *p* value (<0.05). *K*‐means cluster analysis (*K* = 4) was used to group differentially regulated genes in muscle. The number of regulated genes in muscle analysis was 312 (Cluster I), 183 (Cluster II), 1090 (Cluster III), and 1077 (Cluster IV), respectively (Figure S7, Supporting Information). A total of 311 genes were regulated in livers from HFD‐fed PAGR1*^f/f/Hsa‐Cre^* mice, with 162 up‐ and 149 downregulated, respectively (Figure 3e). For pathway analysis, the filtered data sets were uploaded into DAVID Bioinformatics Resources 6.8 to review the biological pathways using the Functional Categories database. The GO analysis was used to interpret data, and the regulated terms ranked by *p* value are shown in Figure S7a,b (Supporting Information). The volcano plot and heatmap analysis of regulated genes were generated by using R software, version 4.0.3, and the ggplot2/gplots package. The raw sequence data reported in this paper were deposited in the Genome Sequence Archive (Genomics, Proteomics & Bioinformatics 2021) in National Genomics Data Center (Nucleic Acids Research 2022), China National Center for Bioinformation/Beijing Institute of Genomics, Chinese Academy of Sciences (GSA: CRA018422 and CRA018416) that were publicly accessible at https://ngdc.cncb.ac.cn/gsa. CRA018422: https://ngdc.cncb.ac.cn/gsa/s/70dho1Xf; CRA018416: https://ngdc.cncb.ac.cn/gsa/s/pRYdEJBi.^[^ ^36^ ^]^

### RNA Analyses

Quantitative reverse transcriptase (RT)‐PCR was carried out following the previously described method.^[^ ^32^ ^]^ In brief, total RNA was extracted from entire gastrocnemius muscle using RNAiso Plus (Takara Bio), and the quality of isolated total RNA was confirmed by ethidium bromide staining. Subsequently, 1 µg total RNA samples were then reverse transcribed using the PrimeScript RT Reagent Kit with gDNA Eraser (Takara Bio). Real‐time quantitative RT‐PCR was performed using the ABI Prism Step‐One system with Reagent Kit from Takara Bio. Specific oligonucleotide primers for target gene sequences are listed in Table S1 (Supporting Information). The target mRNA levels were expressed as arbitrary units and normalized to the expression of *36b4*.

### ChIP, ChIP‐qPCR, and ChIP‐Seq

ChIP assays were conducted in WT or PAGR1*^f/f/Hsa‐Cre^* mice, following previously described methods.^[^ ^16^ ^]^ Briefly, chromatin fragmentation was performed by sonication using a Bioruptor (Diagenode). For PAGR1 ChlP‐seq, H3K27ac ChIP‐Seq, and H3K4me1 ChIP‐Seq, gastrocnemius muscles from three animals were pooled per group. An aliquot of chromatin was precleared with protein G, and immunoprecipitation was conducted with anti‐PAGR1 (ABE1863, Sigma‐Aldrich), anti‐H3K27ac (ab4729, Abcam), and anti‐H3K4me1(ab8895, Abcam) antibodies. Following reversal of cross‐linking, DNA was isolated using the standard phenol–chloroform method. Immunoprecipitated DNA was analyzed by quantitative PCR using the ABI Prism Step‐One system with TB Green Premix Ex Taq II from Takara Bio. The enrichment of genomic regions was calculated relative to input DNA using the standard curve method. Specific oligonucleotide primers for target regions are listed in Table S1 (Supporting Information). For ChIP‐seq, the precipitated DNA samples were amplified using the ChIP Sequencing Sample Preparation Guide provided by Illumina. Beijing Novogene Bioinformatics Technology Co., Ltd. performed deep sequencing using Illumina NovaSeq 6000.

### ChIP‐Seq Data Processing

ChIP‐seq data analysis was conducted following previously published methods.^[^ ^33^ ^]^ Briefly, single end 50 nt reads were mapped to the mouse genome (UCSC mm10) using Bowtie2 (Version 2.2.5). Only sequences uniquely mapped with no more than one mismatch were used as valid reads. The peak caller program MACS2 (Version 2.1.1) was used to identify peaks with the following parameter settings: –keep‐dup = 1, ‐B, –SPMR. Signal pileup tracks in bedGraph format were generated on a per‐million‐reads basis. Genome regions were linked to the gene with the nearest transcription start sites from the UCSC genome browser. Differentially expressed genes from CD‐ and HFD‐muscle RNA‐seq data were integrated with PAGR1 ChIP‐seq data CRA018431to define a set of PAGR1‐directly regulated genes (412 genes). The histone modifications H3K27ac ChIP‐seq dataset (CRA018429), H3K4me1 ChIP‐seq dataset (CRA026606), associated with enhancer activities, were also examined in conjunction with the ChIP‐seq data (CRA018431) for PAGR1. Tag density matrices were calculated using Homer for heatmaps, and visualization was done using Treeview (Version1.1.6r4). A 5 kb region centered on each peak was used with a 10 bp bin size for PAGR1 ChIP‐seq datasets. The heatmap color scale indicated binding signals per million total reads. Genome browser tracks of ChIP‐seq data were visualized using IGV (Version 2.3.70). The raw sequence data reported in this paper were deposited in the Genome Sequence Archive (Genomics, Proteomics & Bioinformatics 2021) in National Genomics Data Center (Nucleic Acids Research 2022), China National Center for Bioinformation/Beijing Institute of Genomics, Chinese Academy of Sciences (GSA: CRA018431 and CRA018429) that were publicly accessible at https://ngdc.cncb.ac.cn/gsa. CRA026606: https://ngdc.cncb.ac.cn/gsa/s/sw6Q07QE; CRA018431: https://ngdc.cncb.ac.cn/gsa/s/ge2b56t5; CRA018429: https://ngdc.cncb.ac.cn/gsa/s/X8eAbou8.^36^

### Histone Extraction

Histone proteins were extracted from skeletal muscle tissues using a standardized acid extraction protocol. Skeletal muscle tissues were thoroughly washed with ice‐cold phosphate‐buffered saline (PBS) to remove blood and debris. Tissues were then resuspended in Triton extraction buffer (PBS containing 0.5% Triton X‐100 v/v, 2 mm phenylmethylsulfonyl fluoride and 0.02% w/v NaN_3_). The homogenized samples were centrifuged to pellet the nuclei. The nuclear pellet was resuspended in 200 µL of 0.2 n HCl and incubated at 4 °C overnight to solubilize histone proteins.

### Antibodies and Immunoblotting Studies

Antibodies directed against GAPDH (60004‐1‐Ig, 1:1000 dilution) were all from Proteintech; antibodies directed against α‐tubulin (bs1699, 1:5000 dilution) were from Bioworld; antibodies directed against p‐AKT Ser473 (#4060, 1:1000 dilution), AKT (#9272, 1:1000 dilution), p‐mTOR Ser2448 (#5536, 1:1000 dilution), p‐mTOR Ser2481 (#2974, 1:1000 dilution), mTOR (#2983, 1:1000 dilution), p‐S6K Thr389 (#9234, 1:1000 dilution), S6K (#2708, 1:1000 dilution), p‐4EBP1 Thr37/46 (#2855, 1:1000 dilution), and 4EBP1 (#9644, 1:1000 dilution) were from Cell Signaling Technology; IRβ (sc‐1:1000 dilution) was from Santa Cruz, Anti‐GLUT4 antibody was a gift of Daniel P. Kelly (Washington University); PAGR1 (#ABE1863, 1:1000 dilution), TBC1D4 (#PLA0289, 1000 dilution) were from Millipore; anti‐H3K27ac (ab4729, Abcam), anti‐H3K4me1(ab8895, Abcam), anti‐H3 (ab1791, Abcam) were from Abcam; antibodies directed against MHC1 (BA‐D5) and MHC2b (BF‐F3) were purchased from the Developmental Studies Hybridoma Bank. Western blotting studies were performed as previously described.^[^ ^34^ ^]^

### Preparation of Subcellular Membrane Fractions from Skeletal Muscle and C2C12 Myotubes

Subcellular membrane fractions were prepared using a modified Hirshman–Grimditch fractionation technique.^[^ ^35^ ^]^ Mouse WV muscles were frozen and powdered in liquid nitrogen, and the resulting homogenate was prepared in homogenization buffer (HB) (50 mm Tris, 150 mm NaCl, 0.5 mm ethylenediaminetetraacetic acid) at 4 °C using a 1 mL Dounce homogenizer. After removing unbroken cells by centrifugation at 500 *g* for 10 min, the homogenate was centrifuged at 20 800 *g* for 20 min at 4 °C. HB buffer was washed for 5 times and the plasma membrane fraction was obtained by centrifugation at 20 800 *g* for 5 min at 4 °C. GLUT4 protein levels were then assessed by Western blot using standard sodium dodecyl sulfate (SDS) sample buffer. C2C12 myotubes were lysed in a detergent‐free lysis buffer by sequential passage through 22 and 27 gauge needles. Unbroken cells were removed via centrifugation at 500 *g* for 10 min, and the plasma membrane was fraction was obtained by centrifugation at 20 800 *g* for 17 min at 4 °C. GLUT4 protein levels were then assessed by Western blot using standard SDS sample buffer.

### Cell Culture and Lentivirus Infection

HEK293T, C2C12, and HepG2 Cells were obtained from the American Type Culture Collection and were cultured at 37 °C and 5% CO_2_ in Dulbecco's modified Eagle's medium (DMEM) supplemented with 10% fetal bovine serum (FBS), 100 U mL^−1^ penicillin and 100 µg mL^−1^ streptomycin. For myocyte differentiation, C2C12 cells were cultured with 2% horse‐serum/DMEM differentiation medium. Lenti‐shPAGR1 (shPAGR1: 5′ GATCCTCAGGAAACAGTCTCT) viruses were generated by cotransfection of 293T cells with the lentiviral vector and two packaging plasmids (psPAX2 and pMD2.G) at a ratio of 2:1.5:1. The virus‐containing supernatants were collected at 48 h posttransfection. The filtered (0.45 µm) supernatants were then used to infect the C2C12 cells. Cells were then incubated in growth media with puromycin, 48–72 h postselection, cells were induced to differentiation for 5 days. For primary white adipocytes isolation and culture, briefly, ≈7–10 days old mice were sacrificed and inguinal adipose tissues were dissected, minced into small pieces, and digested with collagenase D (0.2%). Cells were filtered through 70 µm pores and collected by centrifugation at 1300 *g* for 5 min. Cells were cultured in high DMEM with 10% fetal calf serum (Gibco) and induced to adipocyte differentiation for 2 days with high DMEM containing 10% FBS, 850 nm insulin (Sigma‐Aldrich), 0.5 µm dexamethasone (Sigma‐Aldrich), 250 µm 3‐isobutyl‐1‐methylxanthine (Sigma‐Aldrich), and 10 mm Rosi (Sigma‐Aldrich). After 2 days of culture in high DMEM containing 10% FBS and 160 nm insulin, cells were switched to maintenance medium (DMEM containing 10% FBS) for 4 days before harvesting.^[^ ^29^ ^]^

### Glucose Consumption Assay

C2C12 cells grown in 6‐well plates were differentiated into myotubes for 5 days. Cells were then treated with fresh differentiation medium. At 0, 4, 8, and 12 h postincubation, culture medium was collected from each well and frozen in −80 °C for future glucose measurements using Glucose assay kit (298‐65701, Wako).

### Statistics

GraphPad Prism v9 was used for all statistical analyses. All mouse and cell studies were analyzed by Student's *t* test (2‐tailed) or one‐way ANOVA coupled to a Fisher's LSD post‐hoc test when more than two groups were compared. For datasets that met the assumptions normality and homogeneity of variance, parametric statistical tests were applied. For those that did not satisfy these criteria, nonparametric Mann–Whitney *U* test was employed. No statistical methods were used to predetermine sample sizes, and sample sizes were explicitly stated in the Figure legends. All data points were used in statistical analyses. Data represented the mean ± SEM, with a statistically significant difference defined as a value of *p* < 0.05.

## Conflict of Interest

The authors declare no conflict of interest.

## Author Contributions

C.D., Y.J., L.L., and W.W. contributed equally to this work. C.D., Y.J., L.L., and W.W. designed and performed most of the experiments, analyzed the data, and wrote the paper. D.Z., Z.Z., L.Y., X.C., D.C., Y.M., L.X., C.‐Z.L., Z.‐Y.D., Y.Y., Q.G., Z.S. participated in the collection and analysis of the data. K.G. contributed reagents and provided scientific insight and discussion. T.F., H.‐L.P., and Z.G. conceptualized, interpreted the experiments, and wrote the paper. Z.G. supervised the work. All authors reviewed and contributed to the paper.

## Supporting information



Supporting Information

## Data Availability

The data that support the findings of this study are available on request from the corresponding author. The data are not publicly available due to privacy or ethical restrictions.
