# Supplementary material for: Glucose‐Responsive PAGR1‐Regulated Skeletal Muscle Gene Program Controls Systemic Glucose Homeostasis and Hepatic Metabolism
Source: Adv Sci (Weinh). 2025 Jul 24;12(39):e02763. doi: 10.1002/advs.202502763 (PMC12533408; doi:10.1002/advs.202502763)
Supplement: Supplementary file 1 — Supporting Information [file ADVS-12-e02763-s001.docx]

Supporting Information

**Glucose-Responsive PAGR1-Regulated Skeletal Muscle Gene Program Controls Systemic Glucose Homeostasis and Hepatic Metabolism**

*Chenyun Ding, Yuhuan Jia, Lin Liu, Wen Wang, Danxia Zhou, Zheng Zhou, Likun Yang, Xinyi Chen, Di Chen, Yan Mao, Liwei Xiao, Cai-Zhi Liu, Zhen-Yu Du, Yujing Yin, Qiqi Guo, Zongchao Sun, Kai Ge, Tingting Fu, ^*^ Hai-Long Piao, ^*^ and Zhenji Gan ^*^*

**
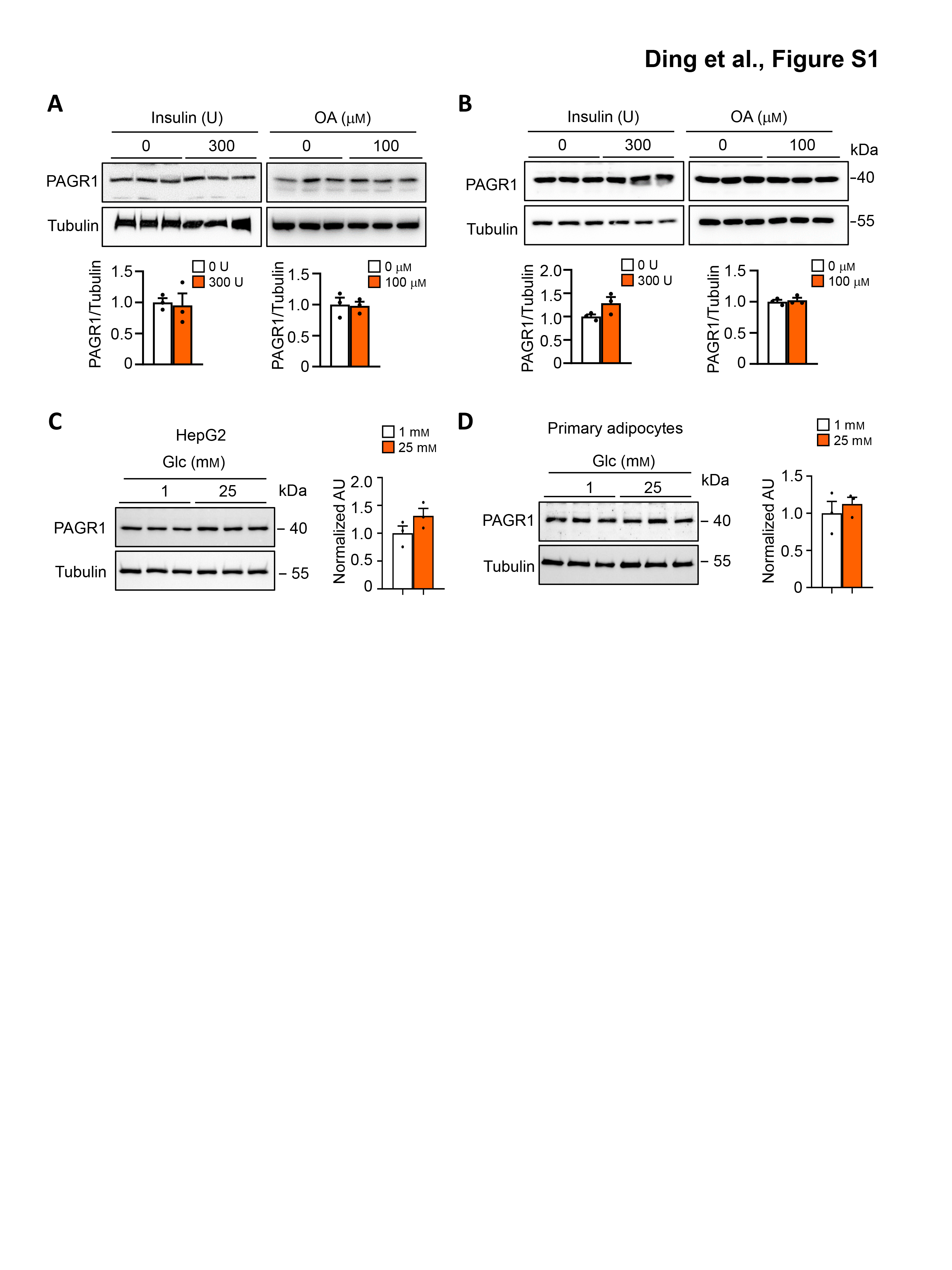
Supplementary Figure S1. Insulin and oleic acid have modest effects on PAGR1 protein levels. (A, B**) Representative Western blot analysis of protein extracts from differentiated C2C12 myotubes and primary human skeletal muscle cells (SkMC) following 12-hour treatments with insulin (Ins) and oleic acid (OA). Quantification of PAGR1 protein levels was normalized to Tubulin and is shown below each blot. *n* = 3 independent experiments. **(C, D**) Representative Western blot analysis of PAGR1 protein levels in differentiated HepG2 cells and primary white adipocytes after 12-hour glucose (Glc) treatment. Tubulin was used as a loading control. Quantification of PAGR1 expression was normalized to Tubulin. *n* = 3 independent experiments. All data are presented as the mean ± SEM. **p* < 0.05 vs. corresponding control groups, as determined by two-tailed unpaired Student’s t-test **(A-D)**.

**
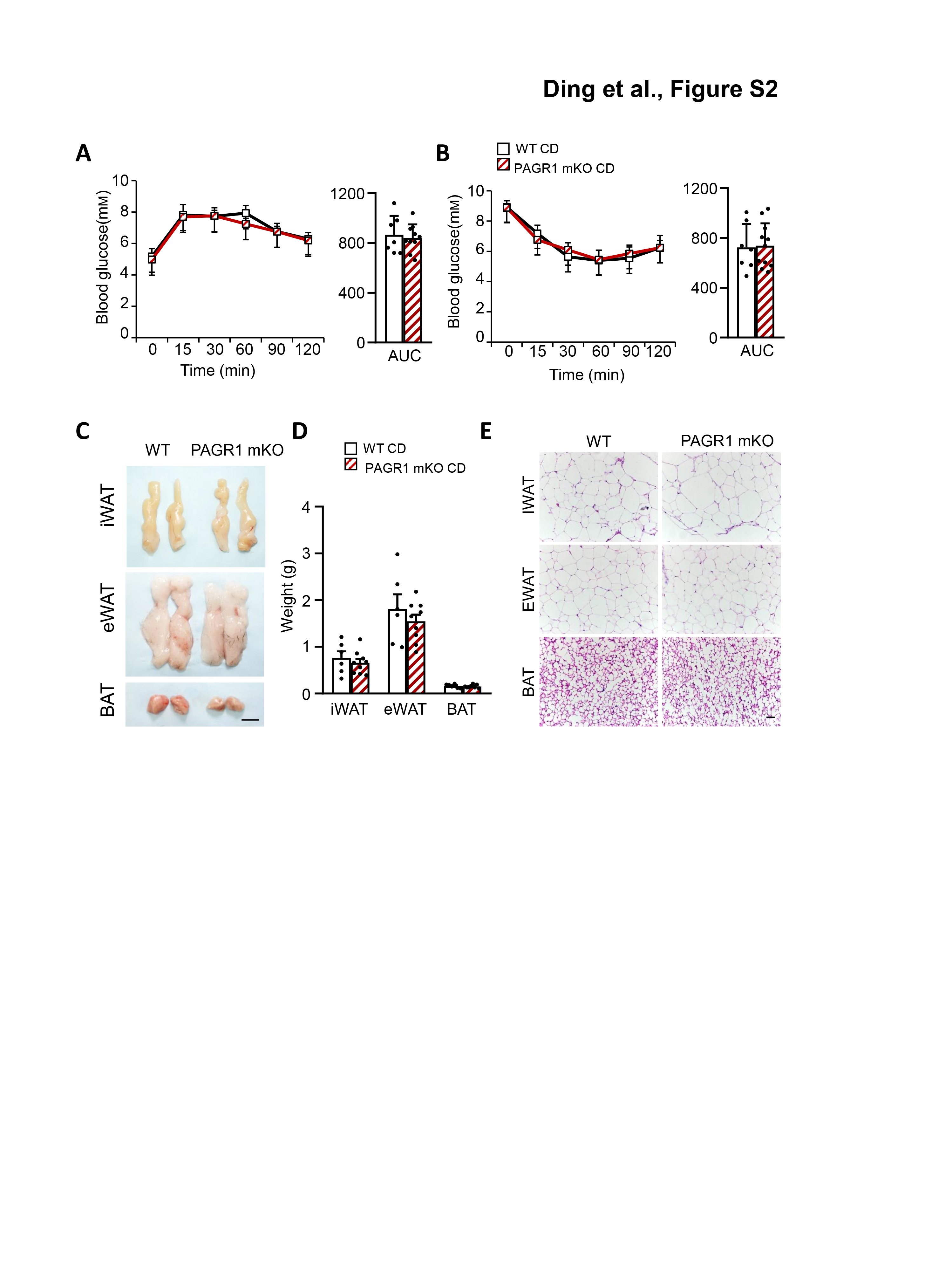
Supplementary Figure S2. PAGR1 mKO mice exhibit comparable body weight and fat mass to WT controls on a chow diet. (A)** Glucose tolerance test (GTT) performed on PAGR1 mKO and WT mice. Right: Quantification of the area under the curve (AUC) for GTT. *n* = 7–10 mice per group. **(B)** Insulin tolerance test (ITT) performed on PAGR1 mKO and WT mice. Right: Quantification of the AUC for ITT. *n* = 7–11 mice per group. **(C)** Representative images of adipose tissue pads from indicated mice fed a chow diet (CD). **(D)** Weight of adipose tissues from PAGR1 mKO and WT mice. *n* = 6-9 mice per group. **(E)** H&E staining of inguinal white adipose tissue (iWAT), epididymal white adipose tissue (eWAT), and brown adipose tissue (BAT) from the indicated mice. Scale bar represents 50 μm. *n* = 4-6 mice per group. All data are shown as mean ± SEM. **p* < 0.05 vs. corresponding WT controls, determined by two-tailed unpaired Student’s t-test **(A, B, D)** and two-way ANOVA **(A, B)** followed by Fisher’s least significant difference (LSD) post-hoc test.

**
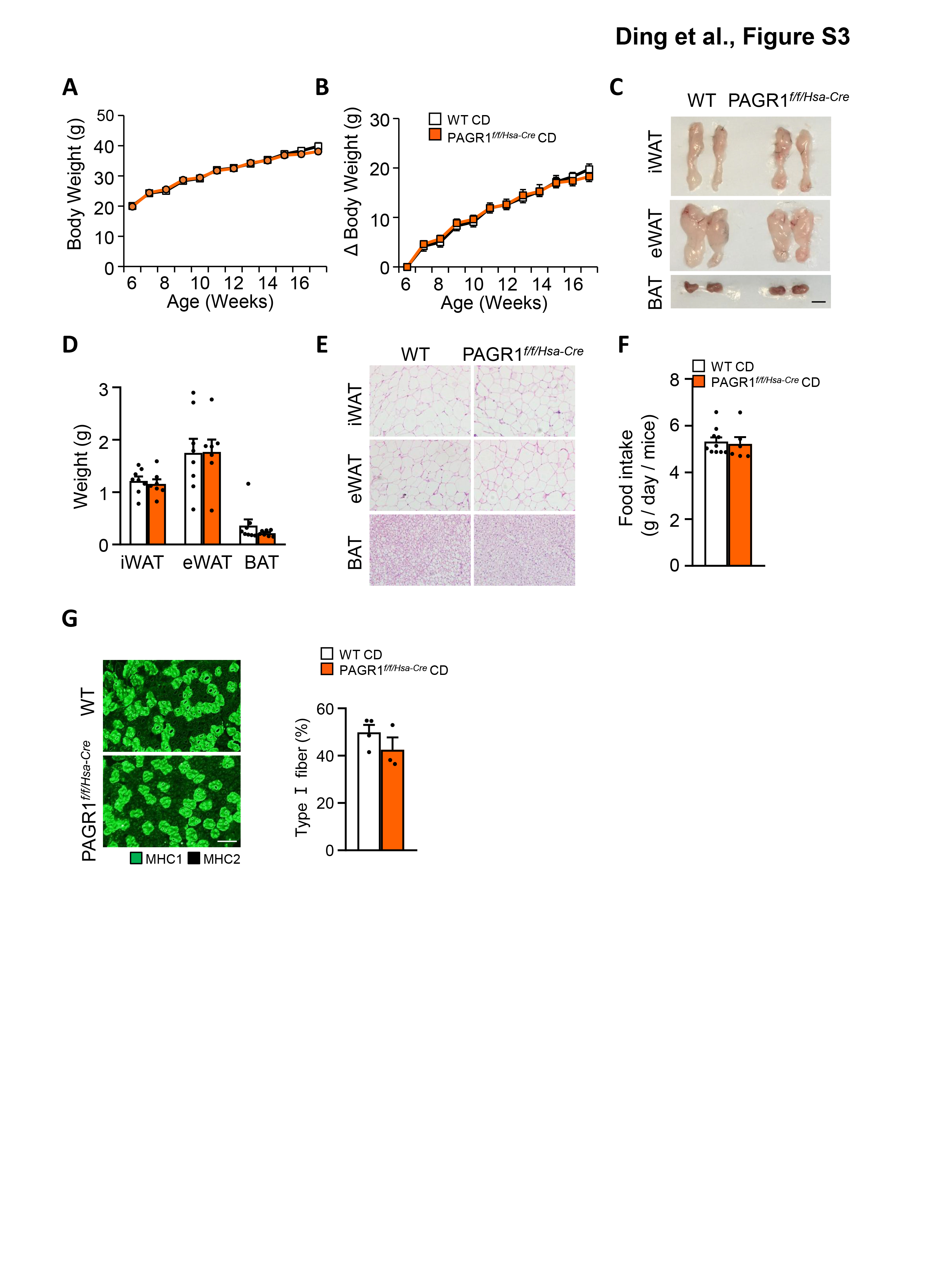
Supplementary Figure S3. PAGR1*^f/f/ Hsa-Cre^* mice exhibited comparable phenotypes to WT controls on a normal chow diet. (A, B)** Growth curves and body weight gain of WT and PAGR1*^f/f/ Hsa-Cre^* mice fed a CD. *n* = 7-9 mice per group. **(C)** Representative images of adipose tissue pads from WT and PAGR1*^f/f/ Hsa-Cre^* mice fed a CD. **(D)** Weights of adipose pads from WT and PAGR1*^f/f/ Hsa-Cre^* mice after 16 weeks on a CD. *n* = 7-8 mice per group. **(E)** H&E staining of inguinal white adipose tissue (iWAT), epididymal white adipose tissue (eWAT), and brown adipose tissue (BAT) from WT and PAGR1*^f/f/Hsa-Cre^* mice fed a CD. Scale bar represents 50 μm. *n* = 3-5 mice per group. **(F)** Daily food intake in WT and PAGR1*^f/f/Hsa-Cre^* mice, expressed as grams consumed per day per mice. *n* = 6-10 mice per group. **(G)** Left: Cross-sections of soleus muscles from WT and PAGR1*^f/f/Hsa-Cre^* mice stained for MHC1 (green). Right: Quantification of the percentage of MHC1-positive myofibers per section. Scale bar represents 50 μm. *n* = 4 mice per group. All data are shown as mean ± SEM. **p* < 0.05 vs. corresponding WT controls, determined by Mann-Whitney test **(D, F, G)** and two-way ANOVA **(A, B)** followed by Fisher’s least significant difference (LSD) post-hoc test.

**
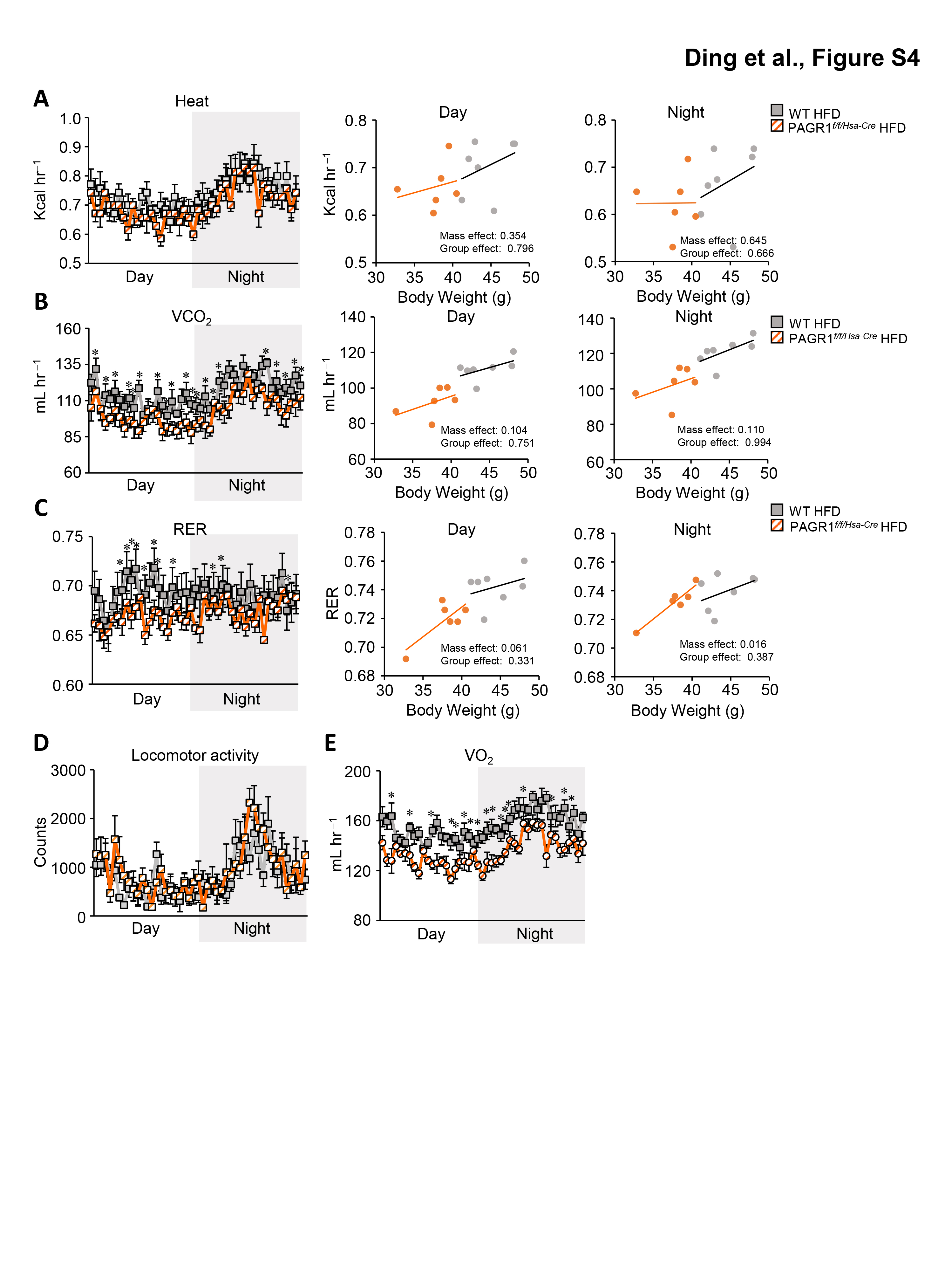
Supplementary Figure S4. Metabolic cage analysis of WT and PAGR1*^f/f/ Hsa-Cre^* mice on HFD. (A-E)** Metabolic evaluation of WT and PAGR1*^f/f/Hsa-Cre^* mice during HFD feeding using a comprehensive laboratory animal monitoring system (CLAMS). Left panels show continuous monitoring of energy expenditure **(A)**, carbon dioxide production (VCO2) **(B)**, respiratory exchange ratio (RER) **(C)**, locomotor activity **(D)**, and absolute oxygen consumption (VO2) **(E)** during light (day) and dark (night) cycles. Right panels display regression plots for each metabolic parameter as a function of body weight in both light and dark cycles. *n* = 6-7 mice per group. All data are shown as mean ± SEM. **p* < 0.05 versus corresponding WT controls, as determined by two-way ANOVA **(A-E)** followed by Fisher’s least significant difference (LSD) post-hoc test.

**
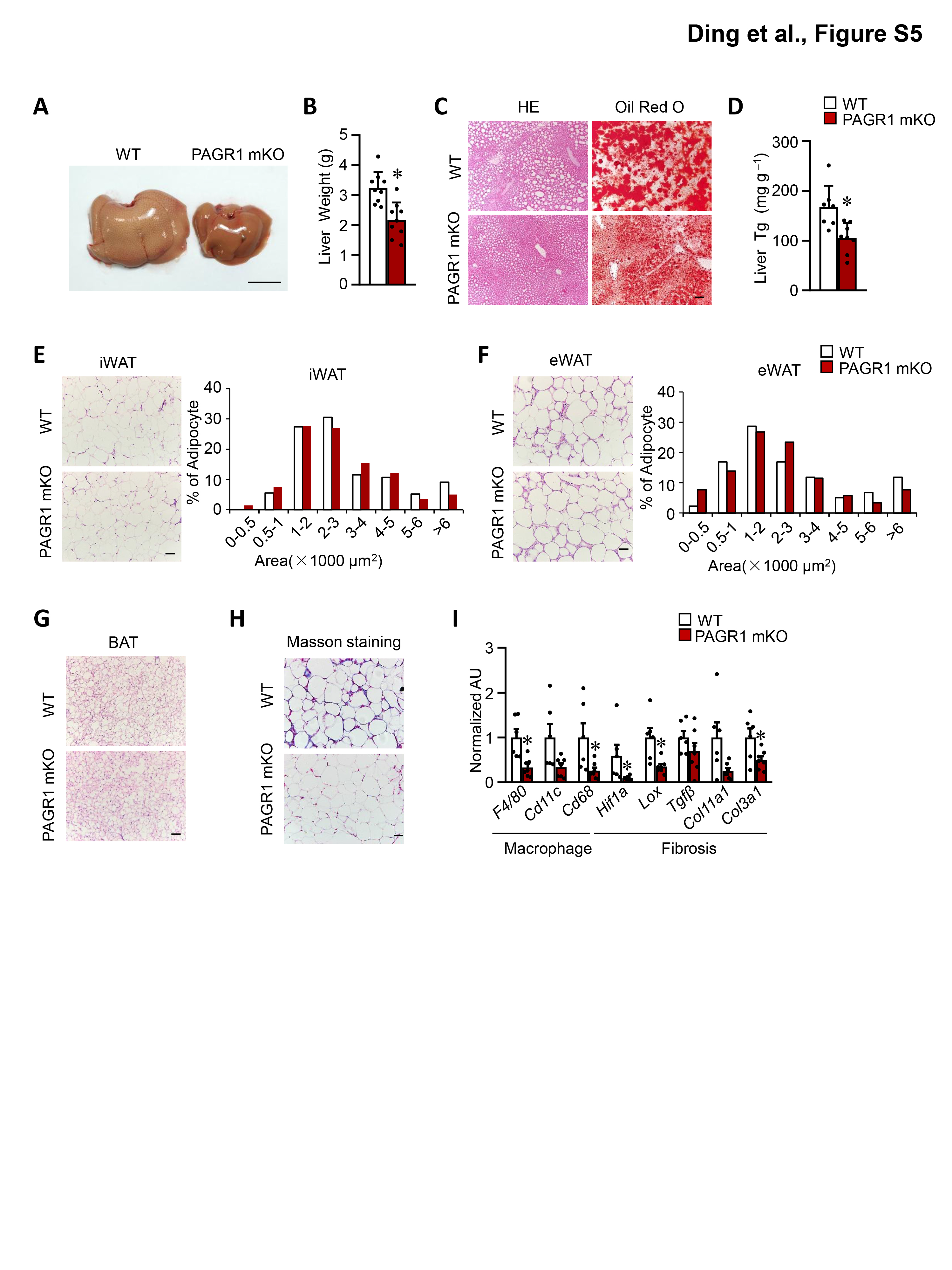
Supplementary Figure S5. Improvement of fatty liver and adipose tissue fibrosis in PAGR1 mKO mice. (A)** Representative images of livers from WT and PAGR1 mKO mice fed an HFD for 16 weeks. **(B)** Liver weights of WT and PAGR1 mKO mice after 16 weeks on an HFD. *n* = 9 mice per group. **(C)** H&E and oil red O staining of liver sections from HFD-fed WT and PAGR1 mKO mice. The scale bar represents 100 μm. *n* = 4 mice per group. **(D)** Liver triglyceride levels in HFD-fed WT and PAGR1 mKO mice. *n* = 7-8 mice per group. **(E-F)** Left: Representative H&E staining of iWAT and eWAT from indicated male mice fed an HFD. Scale bar: 50 μm. Right: Cross-sectional areas of iWAT and eWAT were measured by ImageJ. *n* = 4 mice per group. **(G)** H&E staining of brown adipose tissue (BAT) from WT and PAGR1 mKO mice. The scale bar represents 50 μm. *n* = 3-4 mice per group. **(H)** Masson’s trichrome staining of iWAT from WT and PAGR1 mKO mice, indicating fibrosis levels. The scale bar represents 50 μm. *n* = 3-4 mice per group. **(I)** qRT-PCR analysis of gene expression related to macrophages and fibrosis in eWAT from WT and PAGR1 mKO mice following HFD feeding. *n* = 6-7 mice per group. All data are presented as the mean ± SEM. **p* < 0.05 versus corresponding WT controls, as determined by two-tailed unpaired Student’s t-test **(B, D),** Mann-Whitney test **(I)**.

**
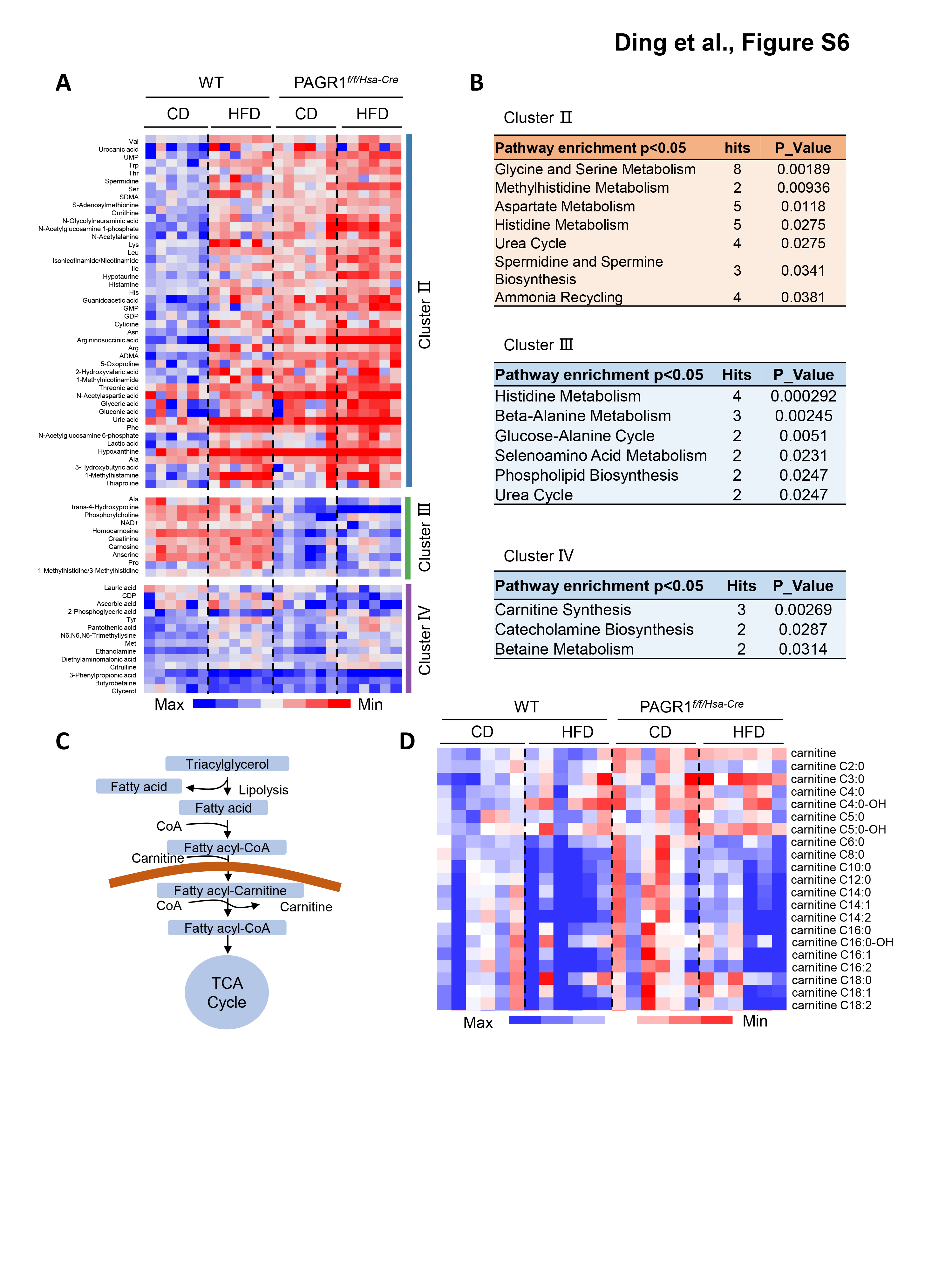
Supplementary Figure S6. Metabolomics analysis of skeletal muscle. (A)** Heatmap showing unsupervised clustering of organic acid and amino acid metabolite content in skeletal muscles from PAGR1*^f/f Hsa-Cre^* mice and WT littermate controls under both chow diet (CD) and high-fat diet (HFD) conditions. *n* = 6 mice per group. **(B)** Metabolite set enrichment analysis (MSEA) of metabolites from clusters II, III, and IV, identifying significantly enriched pathways affected by PAGR1 deficiency. Analysis was conducted using MetaboAnalyst 5.0. **(C)** Schematic representation of glucose and free fatty acid (FFA) metabolism, illustrating the primary energy sources for muscle cells. **(D)** Standardized heatmap showing unsupervised clustering of acyl-carnitine metabolite content in skeletal muscles from PAGR1*^f/f/Hsa-Cre^* mice and WT littermate controls under both CD and HFD conditions. *n* = 6 mice per group.

**
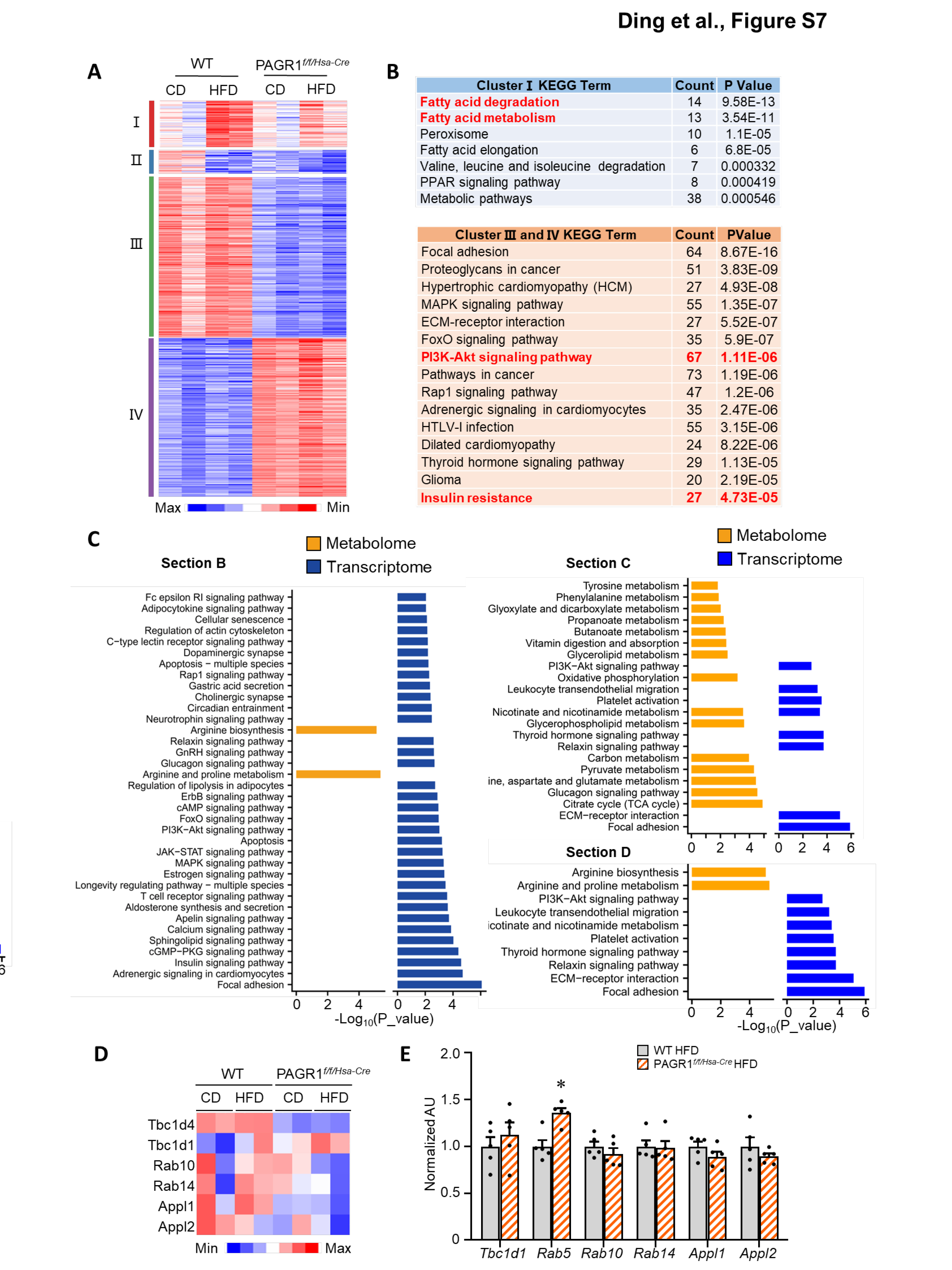
Supplementary Figure S7. Transcriptional analysis of skeletal muscle. (A)** Heatmap analysis of differentially expressed genes in skeletal muscle from WT and PAGR1*^f/f/Hsa-Cre^* mice fed either a chow diet (CD) or high-fat diet (HFD). Each group is represented by RNA-seq data from two independent muscle samples. Genes were clustered into four groups, with a color scheme indicating fold change: red represents a relative increase in expression, while green represents a relative decrease. *n* = 2 independent pools per group. **(B)** GO enrichment analysis of Cluster I (HFD-induced genes suppressed by PAGR1 deficiency) and Clusters III & IV (genes influenced by PAGR1 ablation). The top seven or fifteen enriched terms are shown, highlighting significant biological pathways. **(C)** GO enrichment analysis of integrative analysis of metabolism and transcriptomics in Figure 5A. **(D)** Heatmap showing expression of genes involved in GLUT4 translocation in skeletal muscle from WT and PAGR1*^f/f/Hsa-Cre^* mice. *n* = 2 independent pools per group. **(E)** qRT-PCR analysis of GLUT4 translocation-related genes in skeletal muscle from HFD-fed WT and PAGR1*^f/f/Hsa-Cre^* mice. *n* = 5 mice per group. All data are presented as the mean ± SEM. **p* < 0.05 versus corresponding WT controls, as determined by two-tailed unpaired Mann-Whitney test **(E)**.

**
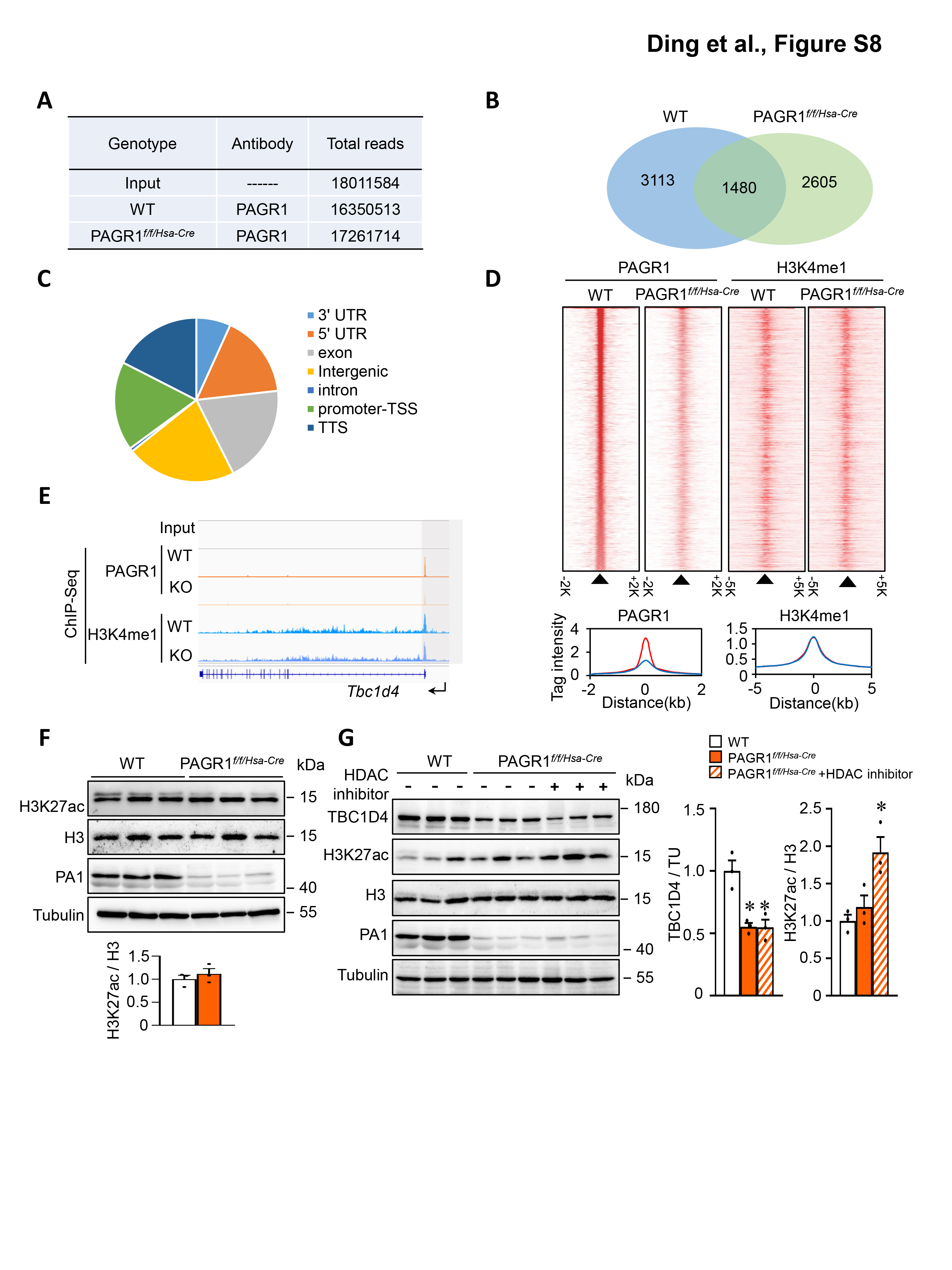
Supplementary Figure S8.** **Cistromic analysis of PAGR1 in skeletal muscle. (A)** Summary of total ChIP-seq reads obtained from chromatin samples of WT and PAGR1*^f/f/Hsa-Cre^* mice enriched with anti-PAGR1 antibody, alongside input control. **(B)** Comparison of PAGR1-binding peaks between WT and PAGR1*^f/f/Hsa-Cre^* mice, identifying 7,955 high-confidence binding regions. The Venn diagram illustrates the shared and unique PAGR1-binding sites in WT (blue) and PAGR1*^f/f/Hsa-Cre^* (green) muscle samples. **(C)** Genomic distribution of PAGR1-binding sites in relation to mouse RefSeq genes. Promoter regions are defined as 1 kb upstream to 200 bp downstream of transcription start sites (TSS). **(D)** (Top) Heatmaps displaying ChIP-Seq signal intensities for H3K4me1 at PAGR1-bound genomic regions in skeletal muscle from WT and PAGR1*^f/f/Hsa-Cre^* mice. (Bottom) Signal intensity profiles of H3K4me1 occupancy, with peaks from WT muscle shown in red and those from PAGR1*^f/f/Hsa-Cre^* muscle in blue. **(E)** ChIP-Seq profiles of PAGR1 and H3K4me1 binding at the *Tbc1d4* gene locus in skeletal muscle. Gray boxes highlight high-confidence PAGR1-binding regions. Input: genomic DNA from skeletal muscle. Gastrocnemius muscles from three mice were pooled per group for PAGR1 and H3K4me1 ChIP-Seq analysis. **(F)** Representative Western blot and quantification of H3K27ac and total H3 levels in skeletal muscle from WT and PAGR1*^f/f/Hsa-Cre^* mice. *n* = 3 mice per group. **(G)** Representative Western blot and quantification of TBC1D4 protein levels in skeletal muscle of PAGR1*^f/f/Hsa-Cre^* mice following intraperitoneal injection of the HDAC inhibitor sodium butyrate (300 mg/kg/day for 5 days) or saline. *n* = 3 mice per group. All data are shown as mean ± SEM. **p* < 0.05 versus corresponding WT controls, as determined by two-tailed unpaired Student’s t-test **(F)** or one-way ANOVA **(G)** followed by Fisher’s least significant difference (LSD) post-hoc test.

**
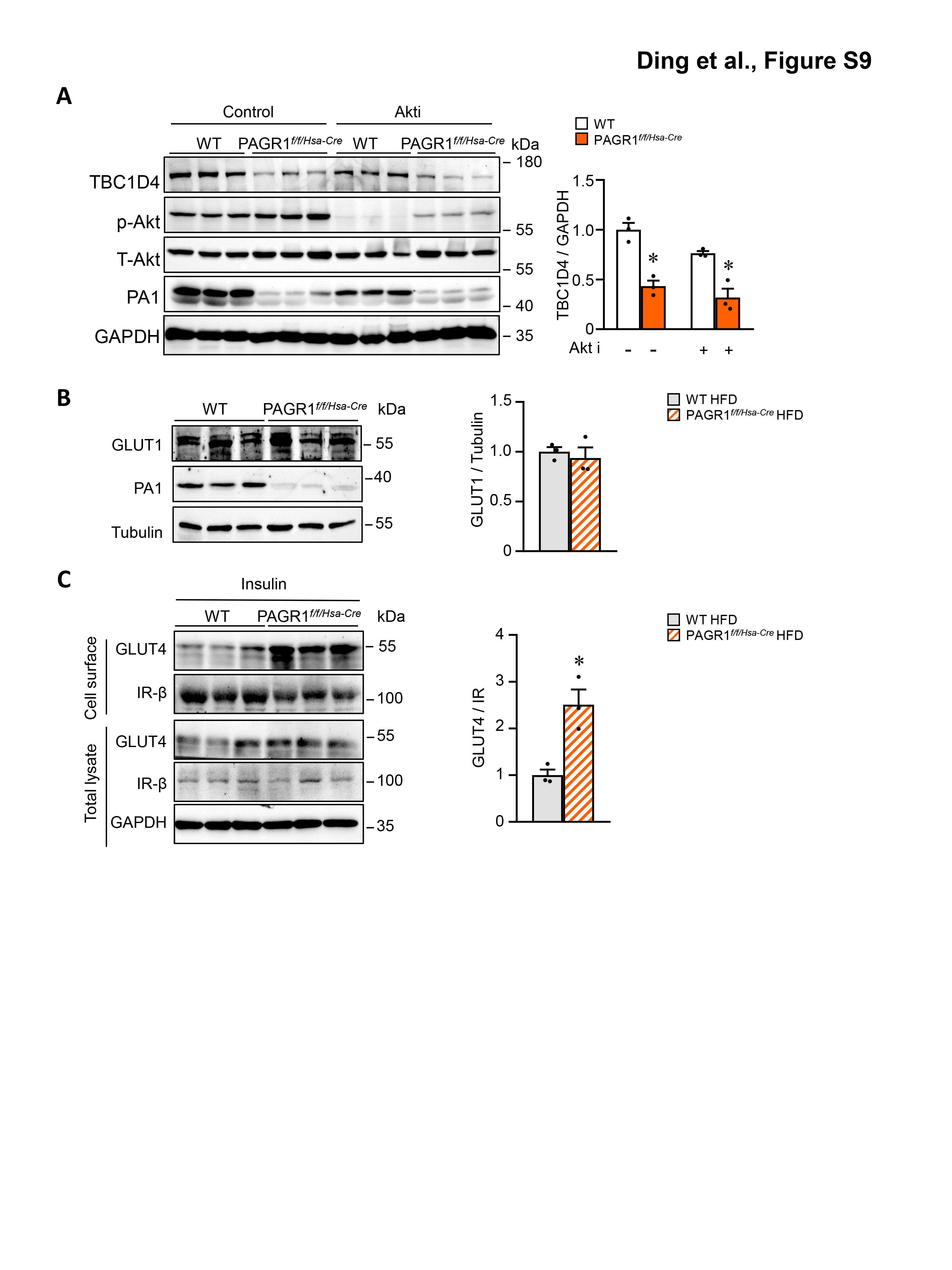
Supplementary Figure S9. Regulation of TBC1D4 expression by PAGR1 in skeletal muscle. (A)** Representative Western blot and quantification of TBC1D4 protein levels in skeletal muscle from WT and PAGR1*^f/f/Hsa-Cre^* mice following intraperitoneal injection of an AKT inhibitor (AKTi-1/2, 50mg kg^−1^ for 2 days) or saline in. *n* = 3 mice per group. **(B)** Representative Western blot analysis of GLUT1 protein expression in GC muscles from WT and PAGR1*^f/f/Hsa-Cre^* mice. *n* = 3 mice per group. **(C)** Cell surface and total GLUT4 protein levels in WV muscles from HFD-fed WT and PAGR1*^f/f/Hsa-Cre^* mice following insulin stimulation. *n* = 3 mice per group. All data are shown as mean ± SEM. **p* < 0.05 versus corresponding WT controls, as determined by two-tailed unpaired Student’s t-test **(A- C)**.

**Supplementary Table S1.**

| **RT-PCR primers** | | |
| --- | --- | --- |
| ***Mouse Gene*** | ***Forward*** | ***Reverse*** |
| *Hk1* | 5’-GAAAGGAGACCAACAGCAGAGC | 5’-TTCGTTCCTCCGAGATCCAAGG |
| *Phka2* | 5’-GTCCAGAGCATTGCTGATGTGC | 5’-ACCAATGTGCCGATACGGTCGA |
| *Pik3r2* | 5'-CAGTACAACGCCAAGCTGGACA | 5'-TGCTGGTGGTAGACCTTGAGCT |
| *Pik3r3* | 5'-ACCACGAGTCTCTCGCTCAGTA | 5'-CCTGATACTGAGAGTGGAACTCC |
| *Pik3r5* | 5'-CTCTGAGCCAAGCATCTCCACT | 5'-GGGTCAAGCATACCGAGGTAGT |
| *Mknk2* | 5'-GAAGGAGCCTATGCCAAAGTCC | 5'-ACAGTGTCTCCACCTCACGGAA |
| *Akt2* | 5'-AGATGAGAGGGAAGAGTGGA | 5'-TGCCTTGTTGACAGCTACC |
| *Sorbs1* | 5'-TACCGAGCGATCGAAAGACT | 5'-AGGAATATCGAGGGGAATGG |
| *Grb14* | 5'-CTGCCAACTGAAGTGCTGTCTC | 5'-GCTGCAAAAGACACCATGTGCT |
| *Cntnap2* | 5'-GTGATGAGACAGGATACAGCGG | 5'-AGTGGTCCACTGCCATCAGGAT |
| *Depdc6* | 5'-TCTCAGGAGACGCATGACAG | 5'-AAAGCTGGTAGATTTCCGACTG |
| *Tbc1d4* | 5'-GAAGGGCCGGCGATTATTTC | 5'-TACTTCCAAGCCGACCTCTC |
| *Acot1* | 5'-AAGAAGCCGTGAACTACCTGCG | 5'-TGTGATGCCCTTCAGGAAGGAG |
| *LPL* | 5'-GCGTAGCAGGAAGTCTGACCAA | 5'-AGCGTCATCAGGAGAAAGGCGA |
| *Dagt2* | 5'-CTGTGCTCTACTTCACCTGGCT | 5'-CTGGATGGGAAAGTAGTCTCGG |
| *Pagr1a* | 5'-GGACACTGGAGGTCCCTCT | 5'-CTGGGAGCTTCTAGTGCCT |
| *F4/80* | 5'-TTTCCTCGCCTGCTTCTTC | 5'-CCCCGTCTCTGTATTCAACC |
| *Cd11c* | 5'-CAGAACTTCCCAACTGCACA | 5'-TCTCTGAAGCTGGCTCATCA |
| *Cd68* | 5'-GCAGCACAGTGGACATTCAT | 5'-TTGCATTTCCACAGCAGAAG |
| *Hif1a* | 5'-CATGATGGCTCCCTTTTTCA | 5'-GTCACCTGGTTGCTGCAATA |
| *Cd36* | 5'-GGACATTGAGATTCTTTTCCTCTG | 5'-GCAAAGGCATTGGCTGGAAGAAC |
| *Scd1* | 5'-GCAAGCTCTACACCTGCCTCTT | 5'-CGTGCCTTGTAAGTTCTGTGGC |
| *Elovl6* | 5'-CGGCATCTGATGAACAAGCGAG | 5'-GTACAGCATGTAAGCACCAGTTC |
| *Tbc1d1* | 5'-TACCACTCCGTGAGCACAGAGA | 5'-GAGTCACAAGCCTTCTGTGGAG |
| *Rab5* | 5'-AAGCACAGTCCTATGCAGATGAC | 5'-GCTGAGTTTGCACCAGGATTCTG |
| *Rab10* | 5'-GGACGATGCCTTCAATACCACC | 5'-GTGATGGTGTGAAATCGCTCCTG |
| *Rab14* | 5'-GCAGATTTGGGATACAGCAGGG | 5'-GGTGAGATTCCTTGCGTCTGTC |
| *Appl1* | 5'-GCACGAGTGAATCAGTCTGCTC | 5'-CTGAACTGCTGGTTCGAGCTGT |
| *Appl2* | 5'-CCGACGATACTGCTTCCAGATC | 5'-TCTGGGTTGTCCGTCAGGTAGA |
| **ChIP-QPCR primers** | | |
| ***Mouse Gene*** | ***Forward*** | ***Reverse*** |
| *Gapdh* | 5’-GCTCTTGAGCTAGATGAGCA | 5’**-**AAGGGCTTGGGAGCACTGTA |
| *Tbc1d4* | 5’-TCAGTTCAAATCACCGGCAC | 5’-GACCTGTCTTGGTTCCTGAA |
